# The nature and prevalence of diversification rate shifts across the Tree of Life

**DOI:** 10.1093/evlett/qrag005

**Published:** 2026-03-03

**Authors:** Bjørn T Kopperud, Alessio Capobianco, John T Clarke, Luis Palazzesi, Sebastian Höhna

**Affiliations:** GeoBio-Center LMU, Ludwig-Maximilians-Universität München, Munich, Germany; Department of Earth and Environmental Sciences, Paleontology & Geobiology, Ludwig-Maximilians-Universität München, Munich, Germany; GeoBio-Center LMU, Ludwig-Maximilians-Universität München, Munich, Germany; Department of Earth and Environmental Sciences, Paleontology & Geobiology, Ludwig-Maximilians-Universität München, Munich, Germany; GeoBio-Center LMU, Ludwig-Maximilians-Universität München, Munich, Germany; Department of Earth and Environmental Sciences, Paleontology & Geobiology, Ludwig-Maximilians-Universität München, Munich, Germany; German Centre for Integrative Biodiversity Research (iDiv) Halle-Jena-Leipzig, Leipzig, Germany; Institute of Biodiversity, Ecology and Evolution, Friedrich Schiller University Jena, Jena, Germany; Department of Palaeobotany, Museo Argentino de Ciencias Naturales & Consejo Nacional de Investigaciones Científicas y Técnicas (CONICET), Buenos Aires, Argentina; GeoBio-Center LMU, Ludwig-Maximilians-Universität München, Munich, Germany; Department of Earth and Environmental Sciences, Paleontology & Geobiology, Ludwig-Maximilians-Universität München, Munich, Germany

**Keywords:** birth–death, diversification, extinction, macroevolution, rate shift, speciation

## Abstract

Strong disparity in species richness among organisms is well documented, but heterogeneity in the underlying diversification process is less understood. Using novel probabilistic methods, we investigate clade-specific diversification rate shifts in several species-rich phylogenies, together representing over 300,000 species across the Tree of Life. We find that diversification rate shifts are extremely prevalent across all clades, with more frequent changes in younger clades and an overall excess of upshifts, resulting in an apparent acceleration of net diversification rates. We also reveal that heterogeneity in diversification rates is related to the tempo of diversification itself. While we find support for more prevalent shifts in speciation rates than extinction rates and more upshifts than downshifts, this is partially due to data insufficiency and inference challenges. Our insights are fundamentally enabled by studying numerous large phylogenies with rigorous statistical methods, showing widespread prevalence of diversification rate shifts across the Tree of Life.

## Introduction

The disparity in species richness across major groups of life is one of the most studied aspects of biodiversity research. Some organisms, including the tuatara reptile, coelacanth fishes, or the ginkgo tree, often nicknamed “living fossils,” represent species-poor groups with only one or a few living taxa despite the old age of their corresponding lineages. Other groups, such as beetles, bats, cichlids, and orchids, are represented by thousands of living species and are much more species-rich than their sister clades or other clades of comparable age. How these stark differences in species richness arise, and if these examples represent exceptions from the general diversification process, remains a significant topic of debate in evolutionary biology (Hedges et al., 2015; Jetz et al., 2012; Sanderson & Donoghue, 1994).

To statistically evaluate differences in species richness, one can obtain diversification rates (i.e., speciation and extinction rates) for different study groups (Sanderson & Donoghue, 1994, 1996). In order to explain the disparity in diversification rates across the Tree of Life, and thus the general process of historical biodiversity, then subclades with differential rates of diversification must have experienced shifts in the rate of diversification, either through many small rate shifts (Budd & Mann, 2025; Davies et al., 2004) or few but large rate shift events (Alfaro et al., 2009; Jetz et al., 2012; Rabosky et al., 2018). Considerable effort has been devoted over the last decades into developing robust and efficient approaches, including the likelihood-based birth–death-shift process, to model lineage-specific diversification rates with diversification rate shifts (e.g., Alfaro et al., 2009; Barido-Sottani et al., 2020; Höhna et al., 2019; Kopperud & Höhna, 2025; Maliet & Morlon, 2022; Quintero et al., 2024; Rabosky, 2014). Speciation events (births) are represented by bifurcations in the phylogeny, increasing the diversity, whereas extinction events (deaths) reduce the diversity of the group, and diversification rate shift events lead to different rates of speciation and extinction of the subtending lineage (Morlon et al., 2024). For example, a shift toward increased speciation rate may lead to the generation of a successful, species-rich clade. In another example, a shift toward a lower speciation rate (and at the same time low extinction rate) may lead to the descendants experiencing few to no speciation events over a long time span. Thus, a shift to lower diversification rates may lead to “living fossils” such as the tuatara or the ginkgo, where one or a few species are separated from their sister groups by hundreds of millions of years.

While several studies have investigated diversification rate variation across the Tree of Life (Jetz et al., 2012; Quintero et al., 2023; Rabosky et al., 2018; Title et al., 2024), these studies were limited in two ways. First, due to computational constraints of the birth–death-shift model (and variants thereof; Barido-Sottani et al., 2020; Höhna et al., 2019; Maliet & Morlon, 2022; Quintero et al., 2024; Rabosky, 2014; Vasconcelos et al., 2022), it has been difficult to investigate species-rich phylogenies (>10,000 taxa). Second, previous studies have been concerned with investigating specific study groups, and less so with the overall pattern of diversification rate heterogeneity as a fundamental process shaping the Tree of Life.

A central research question is whether the number of rate shifts is evenly distributed across the Tree of Life. One hypothesis is that there are some clades with shifts in diversification rates, e.g., rapid radiations, while other clades experienced no diversification rate variation (e.g., Allio et al., 2021; Burress & Muñoz, 2021; Tribble et al., 2024). An alternative hypothesis is that diversification rate variation is not an exception and instead the location of diversification rate shifts across the Tree of Life can be explained by stochastic variation. Thus, the number of rate shifts may simply be related to the time span of the phylogeny, since over a longer time span there have been more opportunities to accumulate rate shifts. When the number of shifts is counted per time unit and per lineage, we can compare phylogenies of different size and age.

Most studies conceptualize diversification rate variation as being driven by phenotypic traits (e.g., Hernández-Hernández & Wiens, 2020; Wiens, 2017). However, no trait that is tested (with the possible exception of size; Cooney & Thomas 2021) is shared across the Tree of Life and therefore specific hypotheses about traits that drive diversification rate variation across the Tree of Life are lacking. Instead, we consider the research question “Are there any fundamental characteristics of clades that experienced more (or fewer) diversification rate shifts?” Here, we focus on two aspects: clade age as this has often been suggested to be linked to species richness (see discussion in Wiens, 2017), and net diversification rate. Regarding our first aspect, an age dependency of diversification rate shifts could indicate some fundamental property of older vs. younger clades or indicate some overall temporal variation in when diversification rate shifts are more prominent. Second, net diversification rates can be seen as an indicator of volatility in terms of phenotypic evolution, as high phenotypic evolvability or flexibility is thought to facilitate adaptation and increase diversification rates (e.g., Onstein, 2020; Peoples et al., 2025). As such, we expect that highly volatile phylogenies (as approximated by high net diversification rates) evolve key innovations (Simpson, 1953) more often than less volatile lineages, in turn resulting in more frequent diversification rate shifts.

We also investigate whether diversification rate shifts led to an upward or downward change in net diversification. Prior empirical evidence suggests that diversification rates in a clade have a tendency to dwindle (Moen & Morlon, 2014; Scantlebury, 2013), although these patterns were found across time and not necessarily among clades. Alternatively, it is possible that older clades could have experienced a rapid radiation due to an upward diversification rate shift event, followed by a gradual decline in diversification rate (Kozak et al., 2006; Lovette & Bermingham, 1999; Rabosky & Lovette, 2008). Importantly, most studies have focused on rapid radiations with an increase in diversification rates. Our study will elucidate whether rapid radiations (i.e., upward shifts) are truly a more common pattern, or whether looking broadly over many large-scale phylogenies shows a more balanced pattern of upward and downward diversification rate shifts.

We test our hypotheses by estimating lineage-specific diversification rates and shifts of diversification rates along lineages. The recently developed Pesto framework (Kopperud & Höhna, 2025) enabled us to fit the birth–death-shift model to numerous large phylogenies obtained from previous publications. Overall, we observe that diversification rate shifts are present in all studied empirical phylogenies. However, some phylogenies experienced far more diversification rate shifts than others. In particular, younger clades appear to exhibit more frequent rate shifts than older clades. Assessing the nature of diversification rate shifts is complicated by intrinsic limitations in detecting such events. Specifically, we can readily detect upshifts in net diversification, but we are less able to detect downshifts, which could explain why we “see” more rapid radiations. Finally, we discuss the prospect of assessing whether a rate shift event is primarily due to a change in extinction rate or speciation rate.

## Methods

### Empirical phylogenies

We compiled a set of 26 published empirical phylogenies. Overall, we targeted species-rich phylogenies that were reconstructed using probabilistic methods, and we selected groups by avoiding taxonomic overlaps (see Supplementary Table S1). Furthermore, we only used trees that had positive branch lengths in units of time (Ma), were ultrametric, and binary splitting. When a publication provided a summary tree from the tree inference procedure, such as a MAP (maximum *a posteriori*) or MCC (maximum clade credibility) summary tree, we used that summary tree. For publications in which a summary tree was not provided, we used the posterior distribution of trees and computed the MCC tree with mean ancestor heights using TreeAnnotator in BEAST (Drummond et al., 2012).

### Modeling clade-specific shifts in diversification rates

We estimated branch-specific diversification rates and diversification rate shifts using the birth–death-shift model (Höhna et al., 2019) implemented in the Julia module Pesto (Kopperud & Höhna, 2025). The birth–death-shift process is a stochastic process with three types of events: speciation events, extinction events, and diversification rate shift events. Two or more events are not allowed to occur at the same time. At a speciation event, the ancestral lineage gives birth to another daughter lineage, which inherits the same speciation and extinction rates. At an extinction event, the lineage simply dies. At a diversification rate shift event, a lineage obtains new speciation and extinction rates that are drawn from a base distribution.

### Discretization of diversification rates

When a rate shift event happens, the new speciation and extinction rates are independent of the ancestral rates. The new speciation and extinction rates follow base distributions (Höhna et al., 2019), where we chose log-normal distributions:


(1) \begin{eqnarray*}
\mathrm{LogNormal}(\log (\hat{\lambda }), 0.587) \\
\mathrm{LogNormal}(\log (\hat{\mu }), 0.587),
\end{eqnarray*}

which have parameters $\hat{\lambda },\hat{\mu }$ controlling the scale, and a standard deviation of 0.587. The standard deviation of 0.587 is chosen such that the 2.5%–97.5% quantile interval spans one order of magnitude on the linear rate scale (e.g., a speciation rate ranging from 0.1 to 1.0). While the birth–death-shift model is conceptually continuous, we use a discrete approximation in order for the model to be mathematically and computationally tractable. Thus, we discretize the log-normal distributions into *n* quantiles and store the middle point of each quantile in a vector $\vec{\lambda } = [\lambda _1, \lambda _2, \dots , \lambda _n]$, and likewise for $\vec{\mu }$. Using all pairwise combinations of $\vec{\lambda }$ and $\vec{\mu }$, we set up two more vectors $\boldsymbol{\lambda }$ and $\boldsymbol{\mu }$, comprised of $n^2 = K$ elements. The vectors $\boldsymbol{\lambda }$ and $\boldsymbol{\mu }$ are the rate categories. The number of rate categories is decided before the model is fitted. In the empirical analyses, we used $n^2=K=100$ rate categories. The rate categories represent the allowed rate variation in the birth–death-shift model. As the number of rate categories increases, the model converges to the full, continuous distribution of diversification rate variation (Kopperud & Höhna, 2025).

### Parameter estimation

The birth–death-shift model has three parameters: $\hat{\lambda }$ controls the mean of the speciation rate distribution, $\hat{\mu }$ controls the mean of the extinction rate distribution, and $\eta$ controls how often the process shifts from one category to another. Using maximum likelihood, we obtain joint estimates of $\hat{\lambda },\hat{\mu }$, and $\eta$. We do this by setting up a series of constraints on the model-fitting procedure, for example, by forcing $\eta$ to be smaller than the other two parameters. We use a numerical optimization algorithm, specifically Newton’s method to iteratively improve the likelihood (Mogensen & Riseth, 2018), until a solution is reached. See the Supplementary Materials for a more detailed description of the optimization procedure.

### Branch-specific rates and rate shift support

When calculating the likelihood of the model, and therefore also when we are fitting the parameters ($\hat{\lambda },\hat{\mu },\eta$), we are integrating over all possible rate shift histories. Thus, the rate shift history does not appear as a parameter in our model. Despite this, the rate shift history is a random, unobserved variable that has a prior distribution (determined by the model) and a posterior distribution (determined by the model and the data). Mapping the posterior distribution of a rate shift history can be done using stochastic mapping (Freyman & Höhna, 2019; Höhna et al., 2019; Huelsenbeck et al., 2003); however, this can be a computational burden (Martínez-Gómez et al., 2024). Instead of using stochastic maps to estimate branch-specific diversification rates, we use a deterministic algorithm (Kopperud & Höhna, 2025). Specifically, we calculate the posterior mean diversification rate per branch as


(2) \begin{eqnarray*}
r = \frac{1}{t_1-t_2}\int _{t_1}^{t_2} \sum _{i=1}^K S_{M,i}(t) (\lambda _i - \mu _i) dt,
\end{eqnarray*}

where $S_{M,i}(t)$ is the marginal probability of the rate category being *i* at time *t* on branch *M*, and *t*_1_ and *t*_2_ are the oldest and youngest times of branch *M*, respectively. $S_{M,i}(t)$ is normalized, meaning that it sums to 1 over the rate categories *i* for any particular time *t*. We calculate the change in the net diversification rate (i.e., the shift size) along a branch as follows:


(3) \begin{eqnarray*}
\Delta r = \sum _{i=1}^K S_{M,i}(t_2) (\lambda _i - \mu _i) - \sum _{i=1}^K S_{M,i}(t_1)(\lambda _i - \mu _i),
\end{eqnarray*}

i.e., as the difference between the means of the oldest and the youngest times of the branch.

In order to assess the statistical support for rate shift events, we use an approximate Bayes factor approach (Shi & Rabosky, 2015). This takes into account both the prior probability ($\pi$) and posterior probability (*P*) of whether or not there was a rate shift event on branch *M*


(4) \begin{eqnarray*}
\mathrm{Bayes factor} = \frac{\frac{P_M(\ge \mathrm{1 shifts})}{\pi _M(\ge \mathrm{1 shifts})}}{\frac{P_M(0 \mathrm{ shifts})}{\pi _M(0 \mathrm{ shifts})}}.
\end{eqnarray*}

We approximate the prior probability $\pi$ by calculating the probability of zero events given a Poisson distribution with rate $(t_1-t_2)\eta$.

In order to calculate the posterior probability *P* of there being no rate shift events on the branch, we solve a set of differential equations—but these are omitted here for brevity (see Kopperud & Höhna, 2025). We consider a branch to have strong statistical support for there being a rate shift event, if the Bayes factor is 10 or higher. To summarize how many branches had strong support across a phylogeny, we use the symbol $N^{*}$ to represent the number of such branches.

Note that the branch rate estimates, as well as the Bayes factors, are given conditionally on the point estimates of $\hat{\lambda },\hat{\mu },\eta$. In effect, we are ignoring estimation error in these parameters. While there is no doubt large uncertainty in our parameter estimates, this omission is a compromise that allows us to obtain branch-specific estimates fast and efficiently even for large trees.

### Validation and exploratory analyses

In the simulation studies performed by Kopperud and Höhna (2025), inference of rate shift events was found to be robust with few false positives. However, the simulation study of Kopperud & Höhna (2025) did not consider the ability to infer rate shift events with respect to the type of rate shift, shift size, or the direction of shift.

Motivated in learning more about whether the method can robustly infer the patterns we found in the empirical data—and to better understand the behavior of the model—we set up a series of auxiliary analyses that are presented in the Supplementary Material. The validation analyses were performed on phylogenies for which we knew the true diversification rate shift history, i.e., simulated data, but we also performed exploratory analyses on empirical data. First, we investigated the inference of upward and downward shifts in diversification, and the ability to detect whether a shift is due to a change in speciation or extinction rate (Supplementary Table S2 and Supplementary Figures S4–S8). Second, we assessed potential methodological biases in estimates of the diversification shift rate, by analyzing simulated birth–death-shift phylogenies of varying tree heights (from 30 to 100 Ma; Supplementary Figures S9–S11). Third, we investigated within-phylogeny estimates of the diversification shift rate (in simulated and empirical phylogenies; Supplementary Figure S12) to see whether it corresponded to the among-phylogeny pattern in empirical phylogenies. Finally, we performed the birth–death-shift analysis on numerous subsets of the ray-finned fish phylogeny (Rabosky et al., 2018) to explore whether subsampling a phylogeny also results in a time scaling pattern (Supplementary Figure S13). We wrote custom scripts to generate the simulated phylogenies, and we used Pesto to infer parameter values and branch-specific diversification rates (see the Supplementary Material for further details).

### Software

We analyzed the phylogenies using Pesto (Kopperud & Höhna, 2025), performed post-processing using R packages for phylogenetic manipulation (Paradis & Schliep, 2019; Wang et al., 2020), and plotted the figures using ggtree (Yu et al., 2017) and Makie.jl (Danisch & Krumbiegel, 2021). We also used TreeAnnotator to create summary trees from posterior distributions of trees (Drummond et al., 2012).

## Results

### Estimating branch rates and rate shift events across the Tree of Life

**Figure 1:**
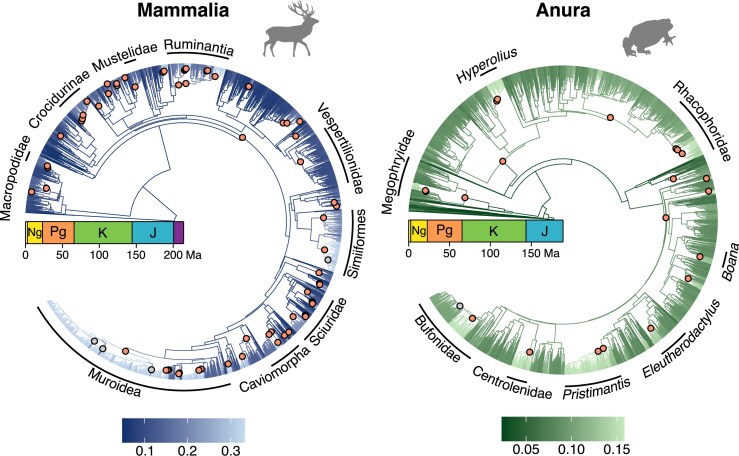
Branch-specific net diversification rate estimates for phylogenies of mammals (Mammalia; Álvarez-Carretero et al., 2022) and frogs (Anura; Portik et al., 2023). Branches are colored according to their mean net diversification rates, expressed in number of events per million year per lineage (see equation 2). The dots (orange for upshift, gray for downshift) represent nodes whose parent branches showed strong support for a rate shift event (Bayes factor $>10$, see equation 4). Some notable clades including at least one strongly supported rate shift event are labeled on the phylogenies. Silhouettes were obtained from www.phylopic.org.

**Figure 2:**
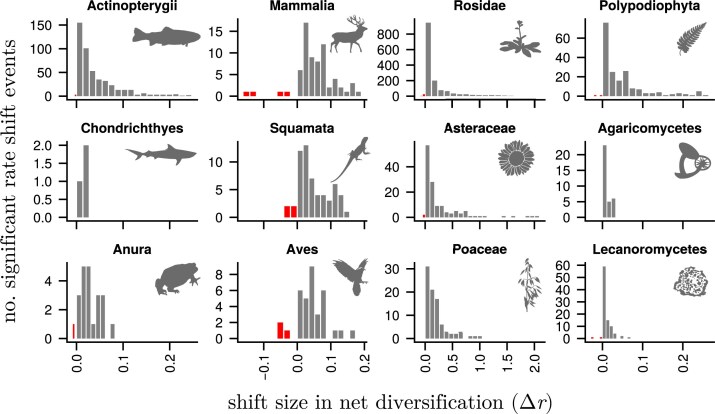
Number of strongly supported rate shift events in selected empirical phylogenies. The number of significant diversification rate shift events ($N^{*}$) is broken down by the branch-specific change in net diversification rate ($\Delta r$, equation 3). Upshifts are in gray and downshifts are in red. The represented phylogenies are ray-finned fishes (Rabosky et al., 2018), cartilaginous fishes (Stein et al., 2018), frogs (Portik et al., 2023), mammals (Álvarez-Carretero et al., 2022), lizards and snakes (Title et al., 2024), birds (tree from Quintero et al., 2022, original analysis by Jetz et al., 2012), rosids (Sun et al., 2020), daisies (Palazzesi et al., 2022), grasses (Spriggs et al., 2014), ferns (Nitta et al., 2022), agaricomycetes (Sánchez-García et al., 2020), and a large group of lichen-forming fungi (Lecanoromycetes; Nelsen et al., 2020). The x-axes are linked for each column. Note that the flowering plants exhibit larger shift sizes than any other groups. See Supplementary Figure S1 for all phylogenies. Silhouettes were obtained from www.phylopic.org.

We assessed heterogeneity in the process of diversification by estimating branch-specific diversification rates and assessing shifts in diversification rates along lineages (Figure 1). When estimating the number of diversification rate shifts, we found that all phylogenies had significant support for lineage-specific diversification rate variation (Figure 2). Specifically, we used approximate Bayes factors to determine whether there was significant support for a rate shift event on a branch (Kopperud & Höhna, 2025; Shi & Rabosky, 2015). Our estimates show that phylogenies experienced a shift in diversification rate ranging from as frequent as every 50, to as rare as every 10,000 million years (in terms of combined branch durations; Supplementary Figure S2b). These results indicate that the process of diversification is heterogeneous across the Tree of Life, i.e., varying diversification rates among lineages are the norm rather than the exception.

### Upshifts vs. downshifts in net diversification

When examining individual phylogenies, we discovered a strong imbalance in terms of whether branch-specific rate shift events resulted in an upward or downward change in net diversification rate. For instance, we inferred 70 strongly supported rate shift events in mammals (Figure 1, left). Of these, 66 were upshifts and only 4 were downshifts. Similarly, we inferred 22 strongly supported rate shifts in frogs, and only one was a downshift (Figure 1, right). Thus, the number of shifts leading to an increase vs. a decrease in net diversification rate appears to be completely imbalanced in favor of increasing net diversification rates.

Overall, we recovered many upshifts and few downshifts across all of the empirical phylogenies we studied (Figure 2, contrary to Harmon et al., 2021; Moen & Morlon, 2014). Taken at face value, a general tendency of ever-increasing diversification rates would lead to an ever more rapidly growing biodiversity (see also Morlon et al., 2012; Rabosky & Benson, 2021). From a paleobiological perspective, however, we believe that downshifts can and do occur. After all, the fossil record tells us that entire clades have gone nearly or completely extinct (e.g., Benoit et al., 2024).

To assess whether the absence of inferred downshifts is due to a methodological bias, we performed several simulation and inference experiments that are presented in the Supplementary Material. Specifically, we simulated several phylogenies that had only one rate shift event, and the rate shift event was either an upward or a downward shift (Supplementary Figures S4–S8). The inferences on the simulated phylogenies revealed that we are able to correctly detect upshifts, and we are able to recover constant diversification. However, when there were downshift events in the true phylogenetic history, we were almost never able to infer the events. This coincides with the statistical level of support for upshifts and downshifts in the empirical analyses. In the rare cases where we recovered downshifts, they were typically recovered with moderately strong support (Bayes factor slightly higher than 10), and almost never recovered with decisive support (Bayes factor higher than 100). In contrast, upshifts were typically recovered with decisive support. While our model allows for diversification rates to decrease, we see that inferred rate shift decreases ($\Delta r < 0$) are usually tiny and spread out over time and across different branches. Coupled with our conservative criterion for assessing whether there was a rate shift event on a branch, this results in a methodological bias where downshifts are almost never detected (Supplementary Figures S7 and S8). This lack of power is not surprising. Our data primarily consist of speciation events, thus giving power to infer upshifts in net diversification. On the other hand, a downward shift in net diversification (either due to lower speciation or increased extinction) is fundamentally difficult to infer from reconstructed phylogenies of only extant taxa, as the signal in the data would be fewer speciation events or more extinction events in the descendant lineages.

### Diversification shift rate across the Tree of Life

We explored whether some phylogenies showed more frequent diversification rate shifts than other phylogenies using two approaches. The first approach is an assessment of the branches in the phylogeny that had strong support for a rate shift event ($N^{*}$). The second approach is an assessment of the shift rate in the phylogeny as a whole (i.e., the shift rate parameter $\eta$). The first approach is more concrete as it corresponds directly to plots of branch-specific net diversification rate estimates such as Figure 1. However, branches without strong statistical support for a rate shift event are also important to consider. Thus, the second approach is useful for assessing diversification rate heterogeneity in phylogenies where there have been many smaller shifts.

**Figure 3:**
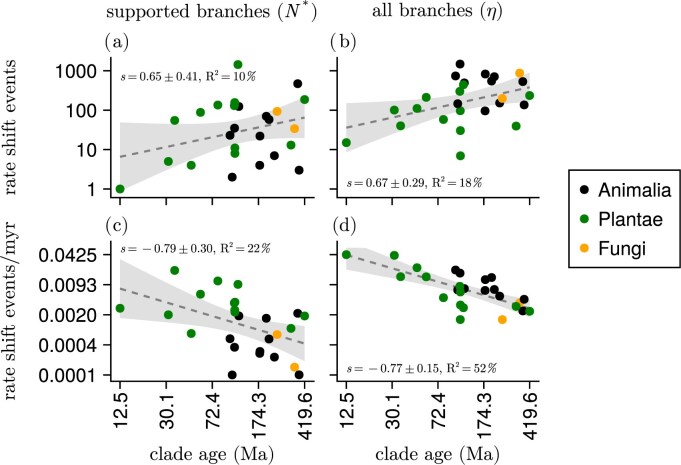
Diversification rate shift events vs. clade age in empirical phylogenies. Panels a and c show the number of branches ($N^{*}$) that showed strong support (Bayes factor $>10$) for there being a rate shift event. Panels b and d show the number of diversification rate shift events according to the shift rate parameter ($\eta$). The clade age is the time from the present until the most-recent common ancestor of the group. The dashed lines are ordinary least-squares regressions on the log-transformed variables (with slope estimate *s*  $\pm$ standard error), and the shaded areas represent two standard error deviations from the line (*R*$^2$ is the amount of variation that is explained by clade age).

A priori, we expect species-rich or older phylogenies to have undergone more diversification rate shifts because they have had more time to accumulate diversification rate shifts than younger phylogenies. We confirm this expectation; older phylogenies display more diversification rate shifts than younger phylogenies (Figure 3a,b). However, our results show a negative age scaling effect in the tempo of diversification rate shifts. Specifically, the number of significant diversification rate shifts per time unit (Figure 3c,d) is negatively correlated with the age of the phylogeny. Most of our younger phylogenies (i.e., originated less than 100 Ma) are plant clades, and these shift their diversification rates more often than older phylogenies (i.e., those that originated at up to 400 Ma) including mostly animal and fungi groups. Specifically, the youngest groups shift their diversification rates about 100 times more often than the oldest groups.

**Figure 4:**
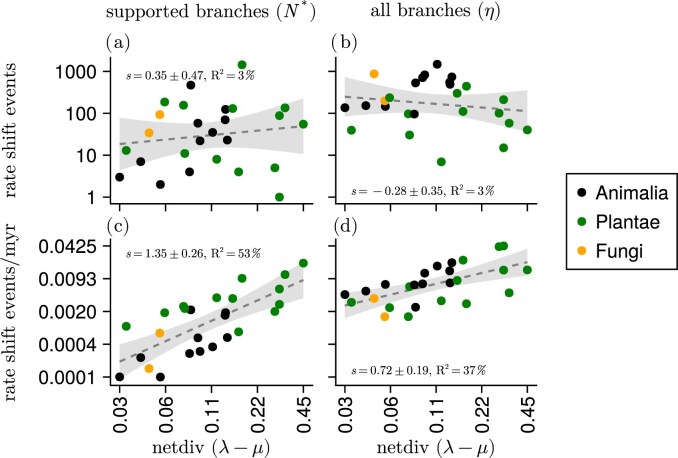
Diversification rate shift events vs. net diversification in empirical phylogenies. Panels a and c show the number of branches ($N^{*}$) that showed strong support (Bayes factor $>10$) for there being a rate shift event. Panels b and d show the number of diversification rate shifts according to the shift rate parameter ($\eta$). The net diversification rate is the average branch-specific rate, weighted by branch length. The dashed lines are ordinary least-squares regressions on the log-transformed variables (with slope estimate *s*  $\pm$ standard error), and the shaded areas represent two standard error deviations from the line (*R*$^2$ is the amount of variation that is explained by net diversification rate).

The number of diversification rate shifts does not appear to be related to the overall net diversification rate (Figure 4a,b). However, we observed that the number of diversification rate shifts per time unit is positively correlated with net diversification rate (Figure 4c,d). In general, plant phylogenies display higher average net diversification rates, and experience diversification rate shifts more often, compared to animals and fungi (Figure 4c).

## Discussion

The empirical phylogenies in our study span many multicellular forms of life, including marine vertebrates, terrestrial animals, flowering plants, ferns, mushroom-forming fungi, and lichen-forming fungi. In total, our dataset includes 11 phylogenies of animals, 13 for plants, and 2 for fungi. Thus, many of the major groups in the Tree of Life are represented, covering a total diversity of over 300,000 species (yet notable missing clades among eukaryotes include coleopterans and other hyperdiverse insect clades, arachnids, crustaceans, molluscs, and orchids). The age of the phylogenies ranges from older groups originating at 420 Ma to radiations as recent as 12 Ma, while the number of taxa represented ranges from about 250 to 20,000. Overall, this representation of major groups covers the process generating historical and present-day multicellular biodiversity, allowing us to learn how variable the process of diversification is.

We found evidence of heterogeneous diversification rates in all empirical phylogenies. Thus, our results indicate that shifts in diversification rates along lineages are prevalent across the Tree of Life. Accounting for among-lineage diversification rate heterogeneity is therefore essential for understanding present and past biodiversity even at moderate taxonomic or temporal scale. Notably, we found that younger phylogenies, as well as clades with higher average net diversification rates, experienced more diversification rate shifts per unit of time. We also found a clear signal of upshifts in net diversification being commonly inferred, while downshifts were not.

Our first general observation revealed that the variability and tempo of diversification are linked (Figure 4). Many phenotypic traits related to diversification (e.g., ecological, functional, or life history traits) are more flexible and evolve more rapidly in groups that also diversify faster (e.g., Cerezer et al., 2023; Folk et al., 2019; Onstein, 2020; Rabosky et al., 2013). In alignment with these ideas, we conjecture that high trait flexibility results in more frequent diversification rate shifts, and therefore unequal species richness across groups. Likewise, if low diversification rates are associated with low trait flexibility, then a less flexible group would have fewer opportunities to evolve phenotypic novelties. In turn, this could result in fewer shifts in the diversification rate, and more evenly distributed species richness.

Our second general observation revealed that younger phylogenies are experiencing more frequent diversification rate shifts (Figure 3). Clade age has often been hypothesized to be correlated with diversification rates (e.g., Henao Diaz et al., 2019; Wiens, 2017). As our estimates show higher rates of diversification rate shifts for younger clades, we argue that older clades have stabilized more and so are undergoing fewer lineage-specific changes impacting diversification rates within the younger parts of their phylogeny.

Moreover, the age scaling effect of the number of diversification rate shifts resembles age scaling effects in other types of evolutionary rates. Notably, age scaling effects have been documented in molecular substitution rates (Ho et al., 2007), rates of morphological evolution (Estes & Arnold, 2007; Hansen, 2024), and diversification rates (Harmon et al., 2021; Henao Diaz et al., 2019). There are several possible methodological explanations for why the age scaling effect could occur. For example, a negative power scaling can arise due to the use of ratios with a common term (Gingerich, 1983; Sheets & Mitchell, 2001), and the effect becomes more pronounced when the range of time intervals being considered is large (De Lisle & Svensson, 2026). Estimation uncertainty in divergence times could also contribute to a spurious age scaling effect (Long, 1980; O’Meara & Beaulieu, 2024). Alternatively, differential model adequacy (i.e., model misspecification) in datasets with short and long time intervals could be an explanatory factor (Kinneberg & Voje, 2026).

In order to evaluate whether the age scaling pattern is a real pattern, as opposed to being indicative of a methodological artefact, we performed several auxiliary analyses that are presented in the Supplementary Material. They show that within-phylogeny diversification shift rates are higher toward the present (Supplementary Figure S12), shift rates in very young phylogenies are overestimated (Supplementary Figure S10a), and subsampled clades from a large phylogeny also exhibit a negative age scaling effect (Supplementary Figure S13). These analyses indicate that the observed empirical age scaling in diversification rate shifts (Figure 3d) can partially be a consequence of the inference procedure employed for this study. However, when looking only at strongly supported shift events, our simulation study shows that the rate ($N^{*}/t$) is consistently underestimated in very young phylogenies (Supplementary Figure S10b), while it is approximately unbiased for older phylogenies. This result goes in the opposite direction of the empirical age scaling pattern in the rate of strongly supported shift events (Figure 3c). Thus, our observed age-scaling effect cannot be explained by methodological causes alone.

A related question is whether a diversification rate shift event is due to a change in the speciation rate, the extinction rate, or both (Donoghue & Sanderson, 2015). In principle, our analyses allow for all three possibilities. In Pesto, it is possible to inspect the posterior mean number of rate shifts, and classify them into the three types of rate shifts (see the Supplementary Material for more details). Our exploration of the inferred type of diversification rate shift revealed two intriguing aspects. First, the number of estimated joint shifts corresponds rather well to the number expected from the prior (Supplementary Figure S3), which in turn is determined by the number of diversification rate categories. This is unfortunate, as the discretization procedure was intended to approximate the birth–death-shift process with continuous rate variation. In principle, as the number of rate categories approaches infinity, the discretized model should converge to the fully continuous model. However, as the number of rate categories increases, the number of joint speciation+extinction shifts converges to the total, while extinction and speciation shifts separately converge to zero (see Supplementary Figure S14). Second, we examined the shifts that resulted in a change in the extinction rate. Even though the prior expectation of speciation and extinction rate shifts is equal, we see in the empirical data that speciation rate shifts are inferred more often than expected, and extinction rate shifts are inferred less often than expected (Supplementary Figure S3). This is in agreement with previous reports of extinction rates being harder or not possible to infer (Beaulieu & O’Meara, 2015; Liow et al., 2010; Nee et al., 1994; Rabosky, 2010). The challenge we see is also similar to Barido-Sottani and Morlon (2025), who found it was difficult to separate whether a rate shift event was due to a change in speciation, extinction, or fossilization rates, or whether there was a combination of shifts.

Our estimates of lineage-specific diversification rates and shifts in diversification rates rely on the birth–death-shift process (Barido-Sottani et al., 2020; Höhna et al., 2019) and specifically its implementation in Pesto (Kopperud & Höhna, 2025). As with any statistical analysis, the assumptions of the underlying model—and any violations of those assumptions—are key to assessing the robustness of parameter estimates. First, our model uses the conventional assumption for taxon sampling, in that every species in the phylogeny has been sampled with equal probability (also called uniform taxon sampling; Höhna, 2014; Höhna et al., 2011). When selecting phylogenies, we aimed for those that had high taxon sampling, while at the same time trying to avoid artificially grafted trees. The mean taxon-sampling fraction was about 45% across the phylogenies, and the lowest was about 11%. Thus, we believe that most of the phylogenetic analyses are robust with respect to taxon sampling. Second, we assume that speciation and extinction rates are allowed to vary by about one order of magnitude within a phylogeny. While this may be a reasonable assumption on short timescales, it is arguably less reasonable for phylogenies on longer timescales (hundreds of millions of years). Third, our model assumes that diversification rates are constant over time within a category. If in reality an upward diversification shift is followed by a concerted slowdown of all lineages within this clade, then our estimates could be biased. Assuming that diversification rates decay within a rate category is possible (Rabosky et al., 2014), yet inferring other types of time-varying patterns is considerably more challenging, and allowing for any type of time-varying function may result in the model not being identifiable (Louca & Pennell, 2020). Fourth, our branch-specific inferences rely on fixed parameter estimates, i.e., the rate of diversification rate shifts $\eta$, the mean speciation rate $\hat{\lambda }$, and the mean extinction rate $\hat{\mu }$. We inferred these parameters using maximum likelihood before estimating diversification rate shifts and lineage-specific diversification rates. Thus, if the shift rate $\eta$ is overestimated, this can lead to an overestimated number of strongly supported diversification rate shifts. For example, broomrapes (Orobanchaceae; Mortimer et al., 2022), daisies (Asteraceae; Palazzesi et al., 2022), and rosids (Rosidae; Sun et al., 2020) had the most extreme estimates with about 0.01 number of strongly supported rate shift events per million years (Figure 3c). However, since all phylogenies were treated equally, we believe that no systematic bias was introduced. Finally, our analyses relied on published phylogenies, which we treated as if they were known without error. Although this is common in large-scale diversification rate analyses (e.g., Quintero et al., 2024), the quality of larger phylogenies might be worse as more ad hoc procedures are required to place taxa and infer divergence times, e.g., placing taxa based on taxonomy without molecular or morphological data (Smith & O’Meara, 2012).

## Conclusions

Biodiversity has changed over time and across lineages. Our results show that net diversification rates vary among lineages for every single analyzed phylogeny. We examined how diversification rates vary across many large-scale phylogenies, spanning major groups of multicellular organisms. Younger phylogenies appear to have experienced diversification rate shifts more often than older phylogenies, similar to how rates of molecular and phenotypic evolution scale with time interval. Furthermore, we found more diversification rate shifts in clades with generally higher diversification rates, indicating general patterns of lability vs. conservatism for both diversification rate shifts and diversification rates themselves. Overall, we inferred many rate shift events where net diversification increased, indicating that rapid radiations are common in contrast to “living fossil” lineages. Understanding whether net diversification rates tend to shift upwards or downwards is complicated by the fact that downward shifts are extremely difficult to detect. Finally, our results provide the foundations for future studies on the nature and prevalence of diversification rate shifts in exceptionally species-rich clades.

## Supplementary Material

qrag005_Supplemental_File

## Data Availability

The data and scripts necessary to reproduce the analyses and the figures are available at https://github.com/kopperud/empirical_shifts.
