# Supplementary material for: The nature and prevalence of diversification rate shifts across the Tree of Life
Source: Evol Lett. 2026 Mar 3;10(2):217–27. doi: 10.1093/evlett/qrag005 (PMC13043920; doi:10.1093/evlett/qrag005)
Supplement: qrag005_Supplemental_File [file qrag005_supplemental_file.pdf]

The nature and prevalence of diversification rate shifts across the Tree of Life

Supplementary Information

Bjørn T. Kopperud[1,2], Alessio Capobianco[1,2], John T. Clarke[1,2,3,4], Luis Palazzesi[5], and Sebastian Höhna[1,2]

[1] *GeoBio-Center LMU, Ludwig-Maximilians-Universität München, 80333 Munich, Germany*
[2] *Department of Earth and Environmental Sciences, Paleontology & Geobiology, Ludwig-Maximilians-Universität München, 80333 Munich, Germany*
[3] *German Centre for Integrative Biodiversity Research (iDiv) Halle-Jena-Leipzig, Leipzig, Germany*
[4] *Institute of Biodiversity, Friedrich Schiller University Jena, Jena, Germany*
[5] *Museo Argentino de Ciencias Naturales & Consejo Nacional de Investigaciones Científicas y Técnicas (CONICET), Buenos Aires C1405DJR, Argentina*

Contents

S1 Supplementary figures and data table . . . 2
S2 Inferring the type of diversification rate shifts using simulated phylogenies . . . 5
S3 Validating the number of rate shift inferences using simulated phylogenies . . . 13
S4 Within-phylogeny estimates of diversification shift rate . . . 15
S5 Assessing the age-scaling effect by analyzing subtrees of the ray-finned fish tree . . . 18
S6 Technical details on estimating the parameters . . . 20
S7 Behaviour with higher rate class discretization . . . 22

S1 Supplementary figures and data table

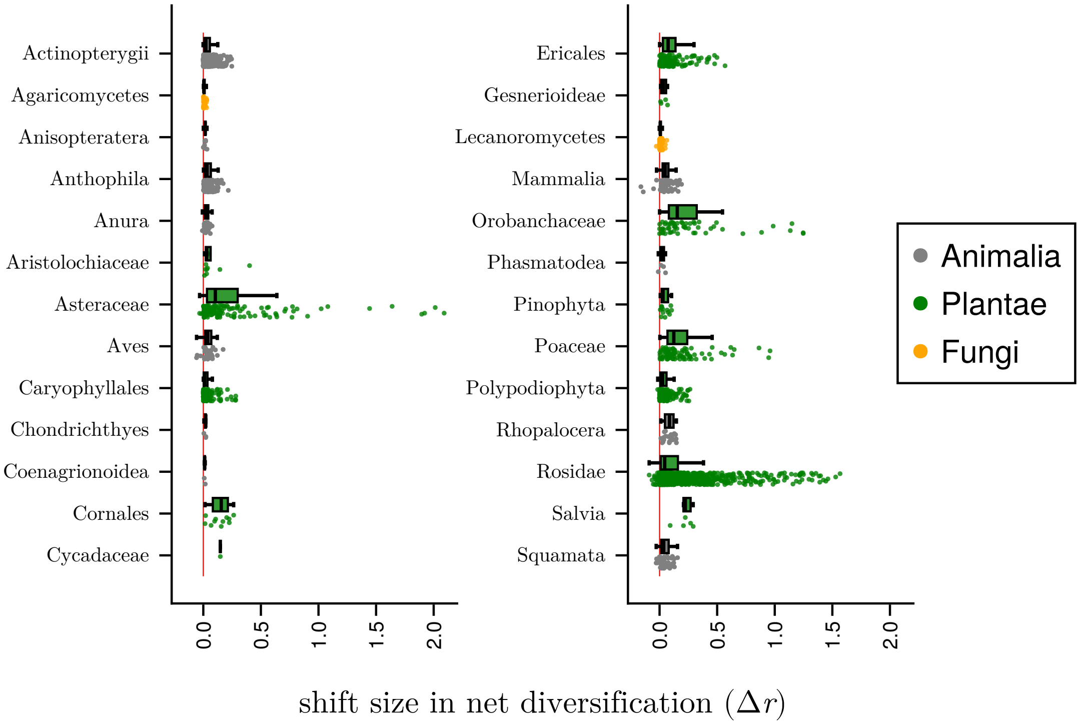

Figure S1: An extended version of Fig. 2, where all of the empirical datasets are included. Each dot represents a branch in the phylogeny that has strong support (Bayes factor > 10) for that there was at least one rate shift event. Note that the majority of shifts are in positive direction (to the right of the red line), however there are some that are negative (to the left of the red line). The box plots show the median, with the crossbar spanning the interquartile range, and the length of the whiskers are 1.5 times the interquartile range.

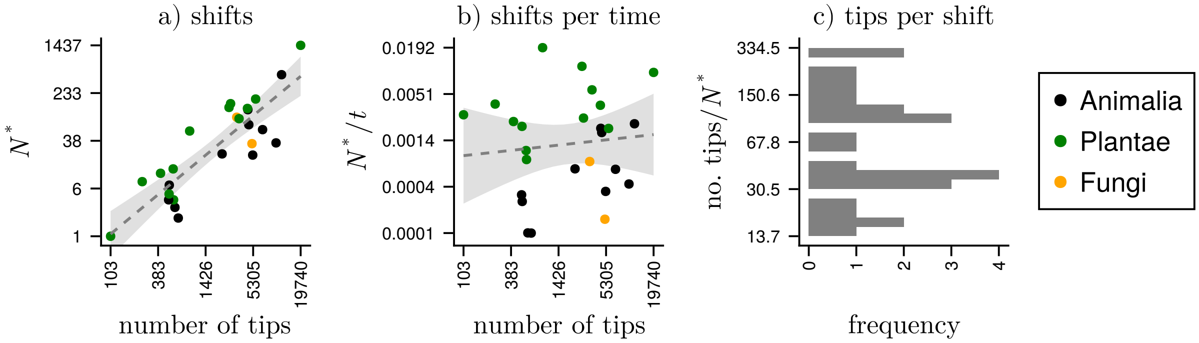

Figure S2: The number of diversification rate shift events, and the number of rate shifts per time (Ma) versus the number of tips included in the reconstructed phylogeny. Each dot in the scatter plots (panels a,b) and each item in the histogram (panel c) represents one empirical phylogeny (see Table S1). The lines are ordinary least-squares regressions, and the shaded areas represents two standard error deviations from the lines.

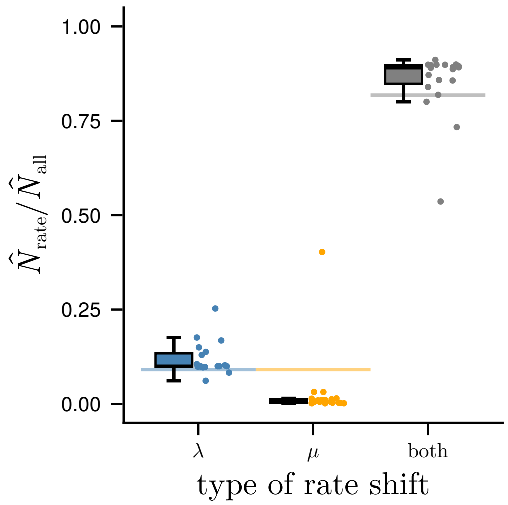

Figure S3: The fraction of number of shifts in speciation rate ($\hat{N}_\lambda$), extinction rate ($\hat{N}_\mu$), and both ($\hat{N}_{\lambda+\mu}$) over all rate shifts ($\hat{N}_{\text{all}} = \hat{N}_\lambda + \hat{N}_\mu + \hat{N}_{\lambda+\mu}$). For more details on the posterior number of rate shift events $\hat{N}$, eq. 12 in Kopperud and Höhna (2025). We summed $\hat{N}_{\text{rate}}$ across the branches that showed strong support for there being a rate shift event (Bayes factor > 10). One empirical phylogeny is represented by three dots (blue, orange, gray), summing to one. We expect *a priori* (horizontal lines) that there are $(n-1)/(n^2-1)$ shifts in a single rate, and $(n-1)^2/(n^2-1)$ joint shifts, or $1/11$ and $9/11$ in this case (since the number of speciation and extinction rate classes was $n = 10$). The box plots show the median, with the crossbar spanning the interquartile range, and the length of the whiskers are 1.5 times the interquartile range. **Bjørn:** need update

Table S1: A summary of the datasets. The age (in millions of years) is the time from the most-recent common ancestor to the present. The sampling fraction is the ratio of species in the tree to the number of described species. The net-diversification rate is the branch-specific net-diversification rate, averaged (but weighted by branch length) over the phylogeny. N^* is the number of branches that showed strong support (Bayes factor > 10) for that there was a rate shift event.

Name	Tax. level	Reference	Age (Ma)	#Taxa	Netdiv	N^*	Sampl.	Sampling reference
Actinopterygii	Class	Rabosky et al. (2018)	368	11638	0.0811	470	0.37	Rabosky et al. (2018)
Agaricomycetes	Class	Varga et al. (2019)	347	5135	0.0429	34	0.26	Varga et al. (2019)
Anisoptera	Infraorder	Letsch et al. (2016)	236	522	0.0378	7	0.17	Letsch et al. (2016)
Anthophila	Unranked	Henríquez-Piskulich et al. (2024)	120	4586	0.138	124	0.23	Henríquez-Piskulich et al. (2024)
Anura	Order	Portik et al. (2023)	179	5242	0.0933	22	0.69	Portik et al. (2023)
Aristolochiaceae	Family	Allio et al. (2021)	111	247	0.120	8	0.40	Mulder (2003)
Asteraceae	Family	Palazzesi et al. (2022)	80	2723	0.341	135	0.12	Palazzesi et al. (2022)
Aves	Class	Quintero et al. (2022)[1]	111	9993	0.113	35	1.00	Quintero et al. (2022)
Caryophyllales	Order	Smith et al. (2018)	111	5036	0.0726	156	0.40	Smith et al. (2018)
Chondrichthyes	Class	Stein et al. (2018)	378	610	0.0274	3	0.51	Stein et al. (2018)
Coenagrionoidea	Superfamily	Willink et al. (2024)	106	669	0.051	2	0.37	Willink et al. (2024)
Cornales	Order	Rose et al. (2018)	111	410	0.074	11	0.68	Thomas et al. (2021)
Cycadaceae	Family	Liu et al. (2021)	13	103	0.313	1	0.88	Liu et al. (2021)
Ericales	Order	Rose et al. (2018)	110	4532	0.154	130	0.36	Rose et al. (2018)
Gesnerioideae	Subfamily	Serrano-Serrano et al. (2017)	44	588	0.168	4	0.49	Serrano-Serrano et al. (2017)
Lecanoromycetes	Class	Nelsen et al. (2020)	249	3373	0.0505	93	0.25	Nelsen et al. (2020)
Mammalia	Class	Álvarez-Carretero et al. (2022)	202	4705	0.137	70	0.70	Upham et al. (2024)
Orobanchaceae	Family	Mortimer et al. (2022)	35	917	0.451	55	0.40	Mortimer et al. (2022)
Phasmatodea	Order	Bank and Bradler (2022)	179	513	0.0796	4	0.15	Bank and Bradler (2022)
Pinophyta	Division	Leslie et al. (2018)	324	578	0.0303	13	0.90	Leslie et al. (2018)
Poaceae	Family	Spriggs et al. (2014)	58	3595	0.313	88	0.29	Spriggs et al. (2014)
Polypodiophyta	Division	Nitta et al. (2022)	420	5685	0.0544	186	0.46	Nitta et al. (2022)
Rhopalocera	Suborder	Kawahara et al. (2023)	101	2244	0.142	23	0.12	Kawahara et al. (2023)
Rosidae	Subclass	Sun et al. (2020)	118	19740	0.177	1437	0.19	Sun et al. (2020)
Salvia	Genus	Kriebel et al. (2019)	31	519	0.292	5	0.52	Kriebel et al. (2019)
Squamata	Order	Title et al. (2024)	213	6885	0.0904	58	0.64	Title et al. (2024)

[1] We obtained the phylogeny tree file from Quintero et al. (2022), however the analysis of inferring the phylogeny was conducted by Jetz et al. (2012).

S2 Inferring the type of diversification rate shifts using simulated phylogenies

We were interested in assessing whether `Pesto` is able to detect what kind of rate shift event that happened on a branch. Specifically, we investigated whether a rate shift event is due to i) a change in the speciation rate, or ii) a change in the extinction rate. In order to test if our inference is able to detect which of the three scenarios that occurred, we simulated several phylogenies where there was exactly zero or one rate shift event. In expectation, the simulation procedure results in a phylogeny that has a moderate "backbone" tempo of diversification. In the subclade that is descended from the rate shift event, it either resulted in an upshift or downshift in net diversification. The specifics of the simulated phylogenies are discussed in detail below.

Table S2: Summary of the models we used to simulate grafted trees (Figs. S4 to S8).

Model	μ	λ	$\lambda - \mu$	age (Ma)	$\mathbb{E}[\#\text{tips}\vert\text{survival}]$
backbone	0.325	0.235	0.09	60	1593.8
upshift (λ)	0.52	0.235	0.285	15	260.7
upshift (μ)	0.325	0.0	0.325	15	261.9
downshift	0.13	0.235	-0.105	40	4.4

$\mathbb{E}[\#\text{tips}|\text{survival}] = 2(1 + \frac{\lambda}{\lambda-\mu}(e^{t(\lambda-\mu)} - 1))$ is the expected number of tips for a phylogeny simulated from a constant-rate birth-death process with birth rate λ, death rate μ, over a time period of t, conditional on that both the left and the right subtrees survived until the present (Höhna, 2015, appendix B). Note that the process begins with a speciation event at the origin, i.e., it has two active lineages from the start.

We simulated the phylogenies using the R-package `TESS` (Höhna et al., 2016) under various settings, see Table S2 for a summary. Specifically, we conditioned on that both the left and right lineages that descended from the root node survived until the present. Using a backbone model with a moderate net-diversification rate ($\lambda - \mu = 0.09$), we simulated 350 trees over a time span of 60 Ma. In each of these 350 backbone trees, we randomly selected a branch that was alive at 15 Ma in the reconstructed tree. At this branch, we pruned the original subtree and replaced it with a separate simulated tree, which we simulated from a model with a higher net-diversification rate. The upshift can either be due to an increase in speciation rate, or a decrease in extinction rate. We selected the change in speciation and extinction rates such that the expected number of tips in the surviving tree was about 260, over the time span of 15 Ma. We performed a similar prune-and-regraft to assess downshifts in diversification, however at an earlier time (at 40 Ma instead of at 15 Ma).

Next, we used `Pesto` to infer branch-specific diversification rates. Ten trees selected at random are plotted in Figs. S4 to S7, and the speciation, extinction and net-diversification rates are mapped with colors on the branches. When there are no diversification rate shift events, `Pesto` tends to correctly infer that there were no shift events Fig. S4. This is expected, as we previously confirmed that `Pesto` has a low false positive rate (Kopperud and Höhna, 2025). For the trees in which there was an upshift event, `Pesto` is usually able to recover the main rate shift event, although not always Figs. S5 and S6. When there was an upshift event, `Pesto` rarely recovers a different upshift as a false positive (e.g., Fig. S5, tree 4). When there was a downshift event, as in Fig. S7, `Pesto` is almost never able to detect it. Thus, we are able to correctly recover i) constancy, ii) upshifts in net-diversification, but not iii) downshift in net-diversification.

Inferring whether the upshift event is due to i) a change in the speciation rate, or ii) a change in the extinction rate, appears to be a more difficult task. We first motivate with color-mapped phylogenies, and follow up with a more quantitative assessment. In some phylogenies (e.g., Fig. S5 trees 1,2), `Pesto` correctly infers that there was a change in the speciation rate. In many other phylogenies (e.g., Fig. S5 trees 3,5,6,8), `Pesto` incorrectly infers that the rate shift event was a joint increase in speciation and decrease in extinction. Similar results can be seen in Fig. S6, where `Pesto` is sometimes but usually not able to correctly infer that the rate shift event was due to a decrease in extinction.

The results for the number of diversification rate shifts across the full 350 simulated phylogenies is summarized in Fig. S8. Here, we make use of the quantity $\hat{N}_M$, which represents the posterior mean number of diversification rate shift events, for a particular branch M. We will recap how to calculate and interpret

$\hat{N}_M$, but see Kopperud and Höhna (2025) for further explanations including how it is derived. Moreover, we specify $\hat{N}_{M,ij}$ for the number of diversification rate change events from rate category j a different rate category i. We use the following differential equation to calculate $\hat{N}_{M,ij}$

$$\frac{d\hat{N}_{M,ij}(t)}{dt} = \begin{cases} -S_{M,j}(t)\dfrac{D_{M,i}(t)}{D_{M,j}(t)}\dfrac{\eta}{K-1} & \text{if } j \neq i \\ 0 & \text{if } j = i, \end{cases} \tag{S1}$$

where $D_{M,i}(t)$ is the probability of observing the clade descended from branch M at time i, given that the process was in rate category i at time t. The quantity $S_{M,j}(t)$ is the marginal probability that the rate category was j at time t on branch M. The initial value for $\hat{N}(t)$ is set to 0 at the oldest time point of the branch, and we use a numerical ODE solver to find a solution at the youngest time point. Solving this for all combinations of i, j gives us a matrix $\hat{\boldsymbol{N}}_M$. Next, we sum over the set of branches $M \in \mathcal{M}$ to obtain

$$\hat{\boldsymbol{N}} = \sum_{M \in \mathcal{M}} \hat{\boldsymbol{N}}_M, \tag{S2}$$

where $\mathcal{M}$ is the set of branches that showed strong statistical support (with Bayes factor > 100, more conservative than in the main text). This gives us a summary of the number of rate change events that can be plotted without the phylogeny. Furthermore, we can decompose $\hat{\boldsymbol{N}}$ and categorize the number of rate shift event by whether it represents a change in the speciation rate ($\hat{N}_\lambda$), the extinction rate ($\hat{N}_\mu$) or a change in both rates simultaneously ($\hat{N}_{\lambda+\mu}$). If we drop the subscripts ij, and use an example with few rate classes (here $n = 2$, $K = 4$), it becomes easier to write the full matrix

$$\hat{\boldsymbol{N}} = \begin{bmatrix} - & \hat{N}_\lambda & \hat{N}_\mu & \hat{N}_{\lambda+\mu} \\ \hat{N}_\lambda & - & \hat{N}_{\lambda+\mu} & \hat{N}_\mu \\ \hat{N}_\mu & \hat{N}_{\lambda+\mu} & - & \hat{N}_\lambda \\ \hat{N}_{\lambda+\mu} & \hat{N}_\mu & \hat{N}_\lambda & - \end{bmatrix}. \tag{S3}$$

Note that the inferences depicted in Figs. S4 to S8 were made using $n = 10$ rate classes. Therefore, the actual $\hat{\boldsymbol{N}}$ matrix in this simulation study has 100 rows and 100 columns.

In order to summarize whether a diversification rate change is due to a change in the speciation or the extinction rate, we use the posterior mean number of diversification rate shifts $\hat{N}_{ij}$ to construct three summary metrics. The metrics describe the net change in the rate (speciation, extinction or net-diversification), weighted by the number of rate change events

$$\begin{aligned} \Delta\lambda &= \sum_{i,j} (\lambda_i - \lambda_j)\hat{N}_{ij} \\ \Delta\mu &= \sum_{i,j} (\mu_i - \mu_j)\hat{N}_{ij} \\ \Delta r &= \sum_{i,j} (r_i - r_j)\hat{N}_{ij}, \end{aligned} \tag{S4}$$

where $r_i = \lambda_i - \mu_i$ represents the net-diversification rate in rate category i. The results are depicted in Fig. S8. Each simulated phylogeny represents a count in the histogram, and the vertical dashed line represents the true change in each rate.

There are five striking results. First, the diversification-rate changes in the constant-rate backbone trees are inferred almost perfectly, with only a handful of false positive upshifts in net diversification. Second, the downshifts are never recovered correctly — the trees that include downshifts appear as if they have been simulated by a constant-rate process. Third, the upshift scenarios (both due to changes in μ and λ) appear to be somewhat recovered, if we consider the inferred change in net-diversification (Δr, Fig. S8, right column), although there is considerable estimation error. Fourth, it seems to be very difficult to infer whether a rate shift event was due to a change in the speciation or the extinction rate. For the scenario where the true upshift was due to a change in the speciation rate, the estimates appear to be approximately centered around

the true change (vertical dashed line). When the true upshift was due to a change in the extinction rate (third row), however, there is a mismatch between the inference and the true change. While the inferred change in speciation has the correct mode ($\Delta\lambda = 0$), the inferred change in extinction is strongly biased. As a fifth point, we do not interpret these results as it being impossible to detect whether the upshift was due to a change in the speciation or the extinction rate. If the patterns in the data were identical, we would expect to see equal or very similar distributions in the second and third rows in Fig. S8. The difference can not be attributed to difference in total species-richness, as we conditioned both grafted in-groups to have about 260 expected number of tips. As the distributions are clearly different, we think that there is some signal available in the divergence times that gives information about the nature of the diversification rate shifts.

Telling whether an upshift is due to a change in the speciation or extinction rate is no doubt a difficult task. The ability of inferring which rate that shifted is perhaps limited by the size of the phylogeny. It may be the case that we need more species-rich trees to reliably infer the correct type of rate shift events (these trees had on average about 2000 tips). However, our results could also be driven by the model assumptions. In the birth-death-shift model that we set up, we implicitly assumed that the going from any rate category j to any other rate category i is happening at an equal rate. This implies i) that the rate of speciation and extinction shifts are equal, but also that ii) joint speciation+extinction rate shifts are more common than single shifts, and the ratio increases with the number of rate categories. We envision that the overall hypothesis could be addressed more elegantly if we i) allowed the rate of speciation and extinction shifts to be different, and ii) disallowed joint shifts entirely. Nevertheless, assessing whether a rate shift event is due to a change in the speciation or the extinction rate requires further research.

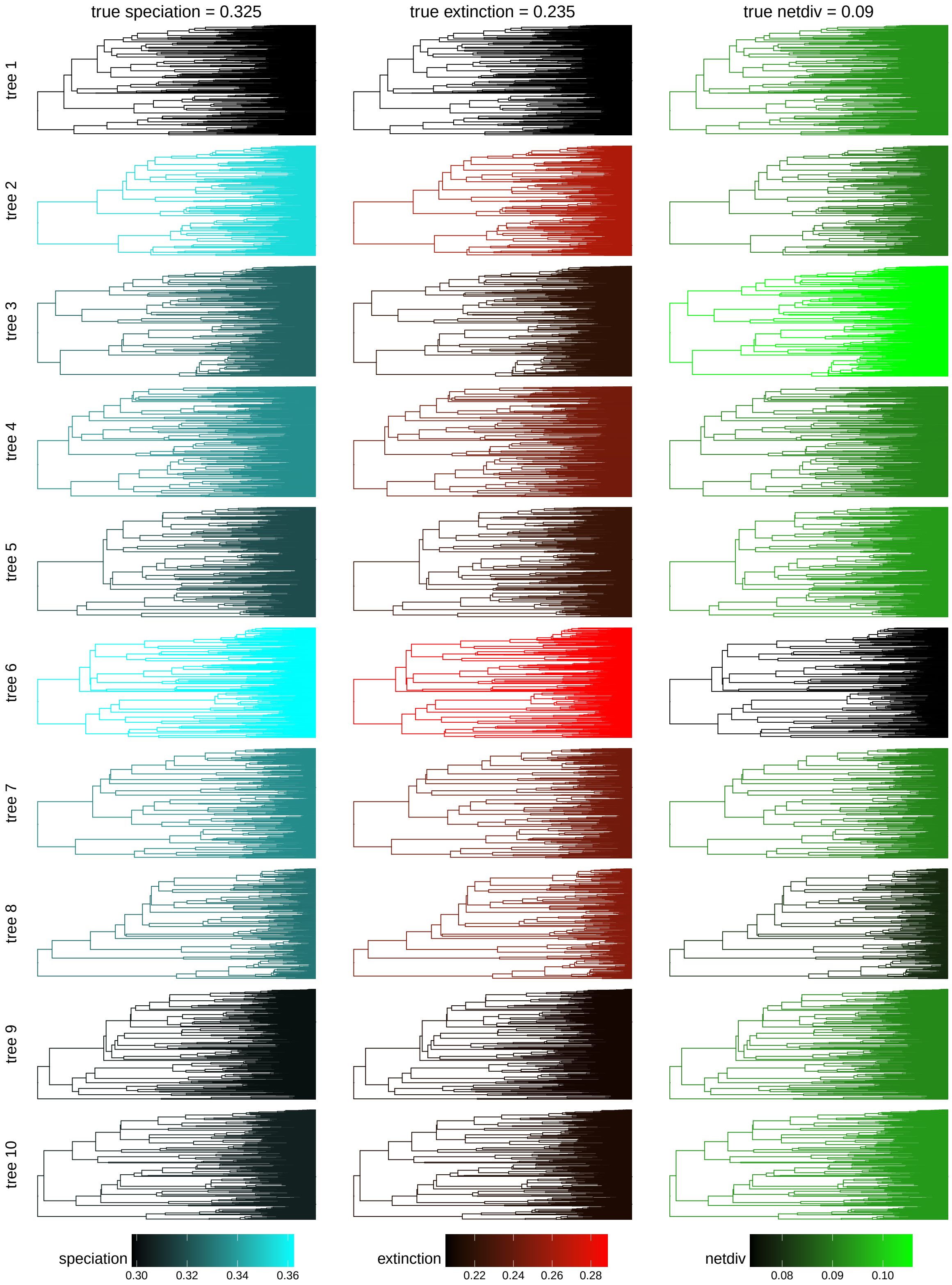

Figure S4: Ten trees where we simulated a backbone tree under the constant-rate birth-death model ($\lambda = 0.325, \mu = 0.235$) for a time period of 60 Ma. We inferred branch-specific speciation, extinction and net-diversification rates (shown in color). There is some estimation error in the rates, but we almost never recovered a rate shift event (low false positive ratio).

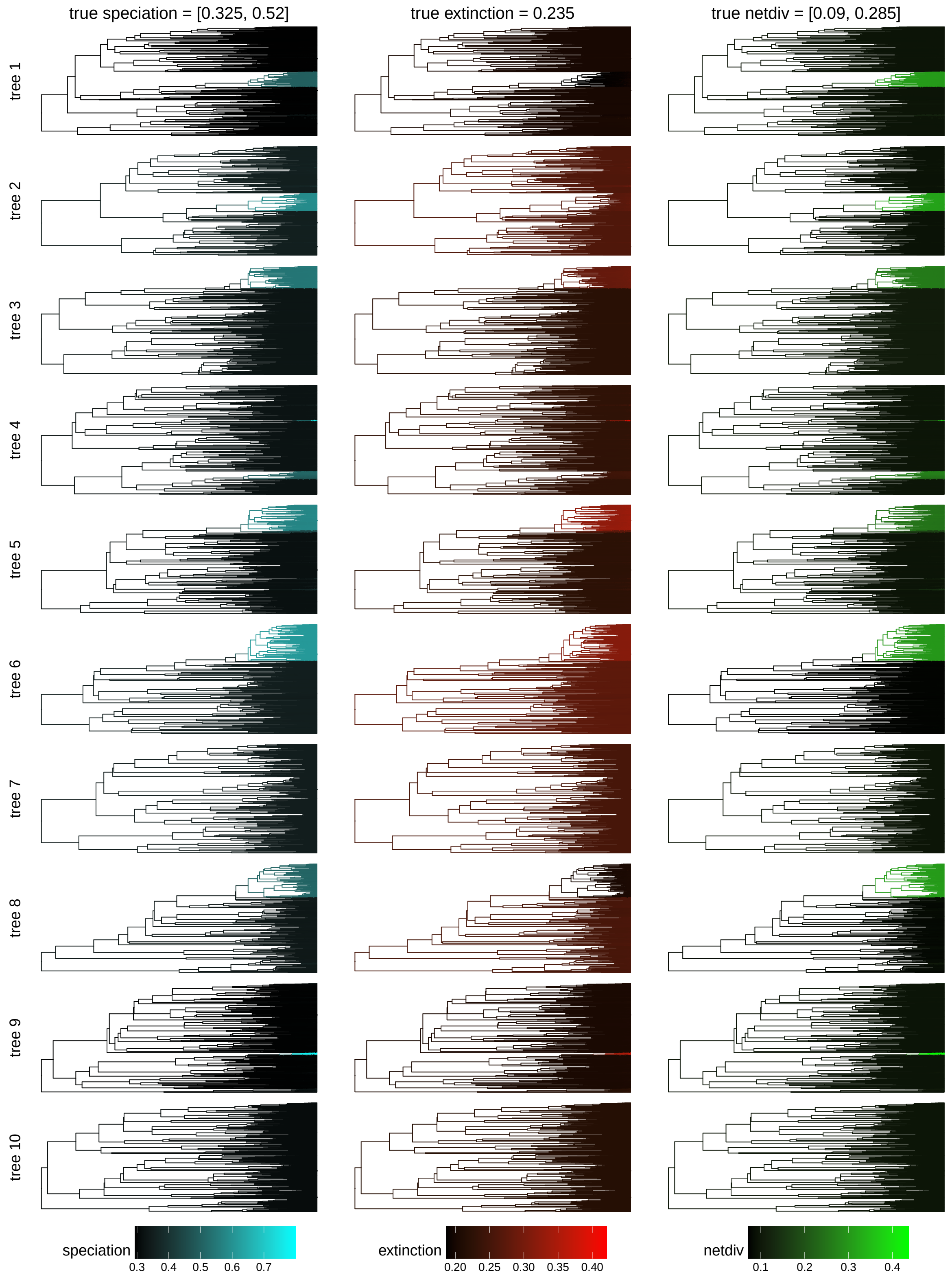

Figure S5: Ten trees where we simulated a backbone tree under the constant-rate birth-death model ($\lambda = 0.325, \mu = 0.235$) for a time period of 60 Ma, and replaced a randomly selected subtree at 15 Ma with a tree that had a higher speciation rate ($\lambda = 0.52$). We inferred branch-specific speciation, extinction and net-diversification rates (shown in color). The shift in net-diversification rate is usually recovered, but not always. When a shift in speciation rate occurs, it can often be mis-identified as a shift in the extinction rate, or as a joint shift (speciation and extinction rate).

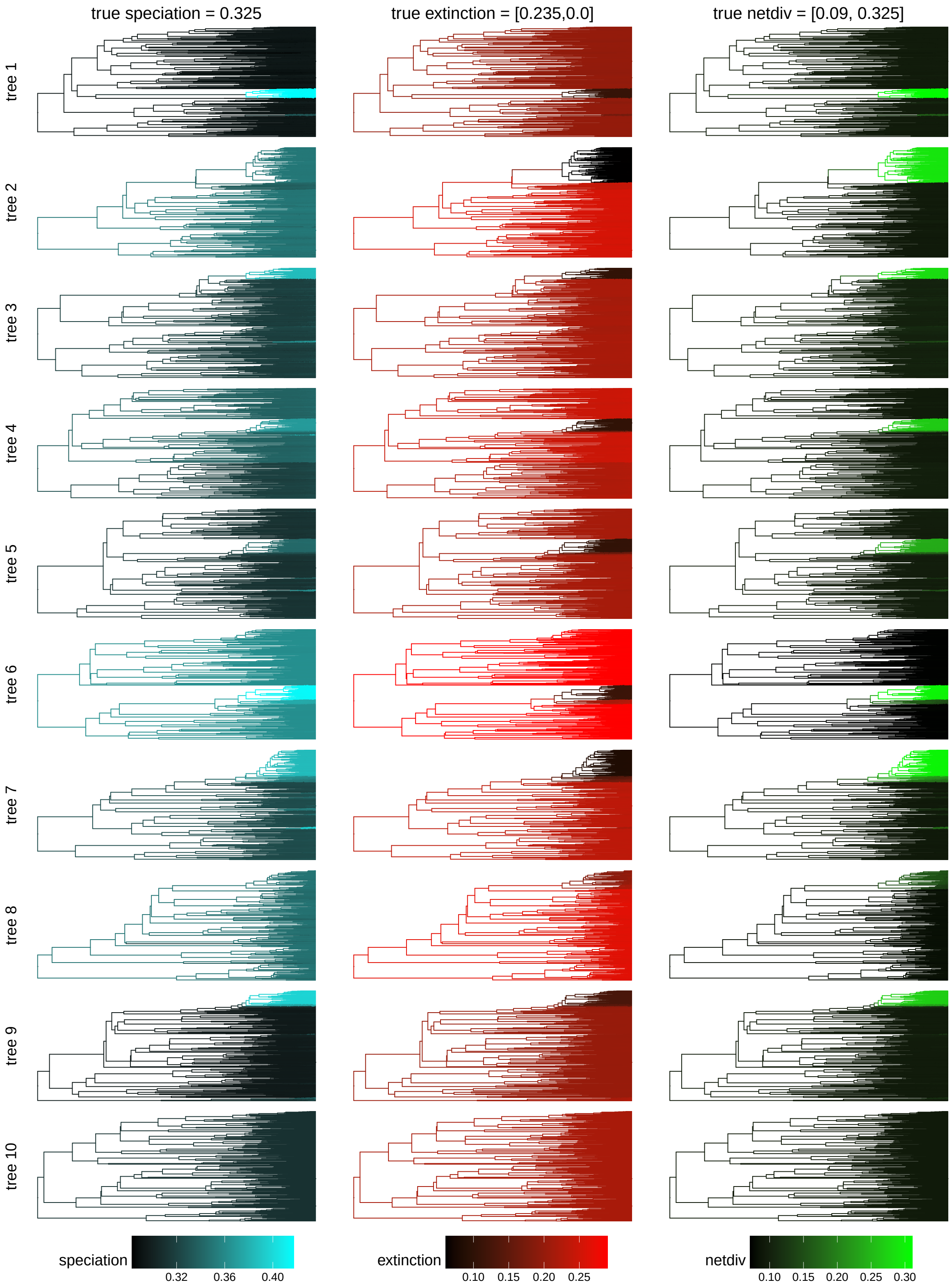

Figure S6: Ten trees where we simulated a backbone tree under the constant-rate birth-death model ($\lambda = 0.325, \mu = 0.235$) for a time period of 60 Ma, and replaced a randomly selected subtree at 15 Ma with a tree that had a lower extinction rate ($\mu = 0.0$). We inferred branch-specific speciation, extinction and net-diversification rates (shown in color). The shift in net-diversification rate is usually recovered, but not always. When a shift in extinction rate occurs, it can often be mis-identified as a shift in the speciation rate, or as a joint shift (speciation and extinction rate).

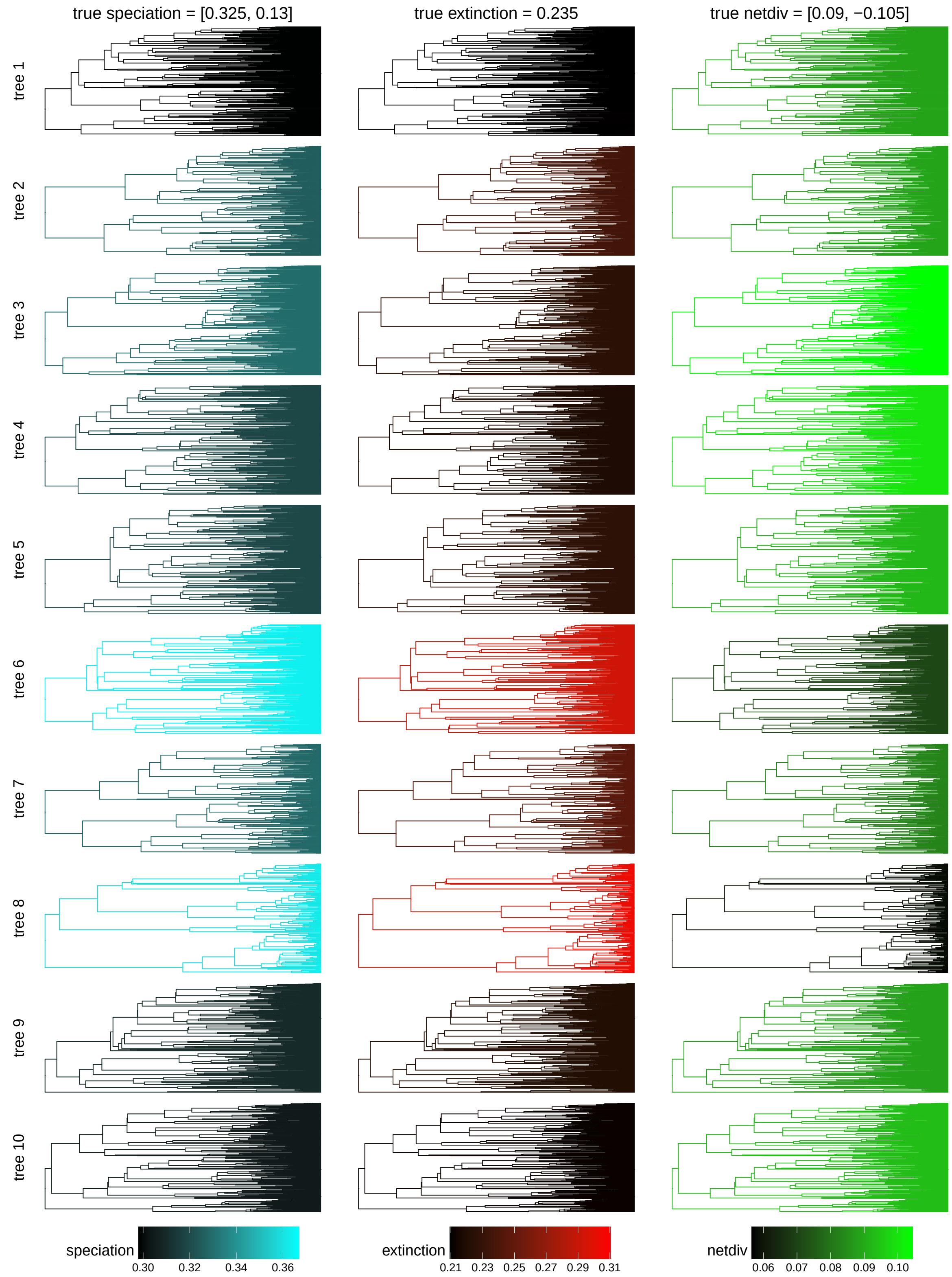

Figure S7: Ten trees where we simulated a backbone tree under the constant-rate birth-death model ($\lambda = 0.325, \mu = 0.235$) for a time period of 60 Ma, and replaced a randomly selected subtree at 40 Ma with a tree that had a lower speciation rate ($\lambda = 0.13$). We inferred branch-specific speciation, extinction and net-diversification rates (shown in color). The rate shift event that leads to a reduction in net-diversification rate is virtually never recovered.

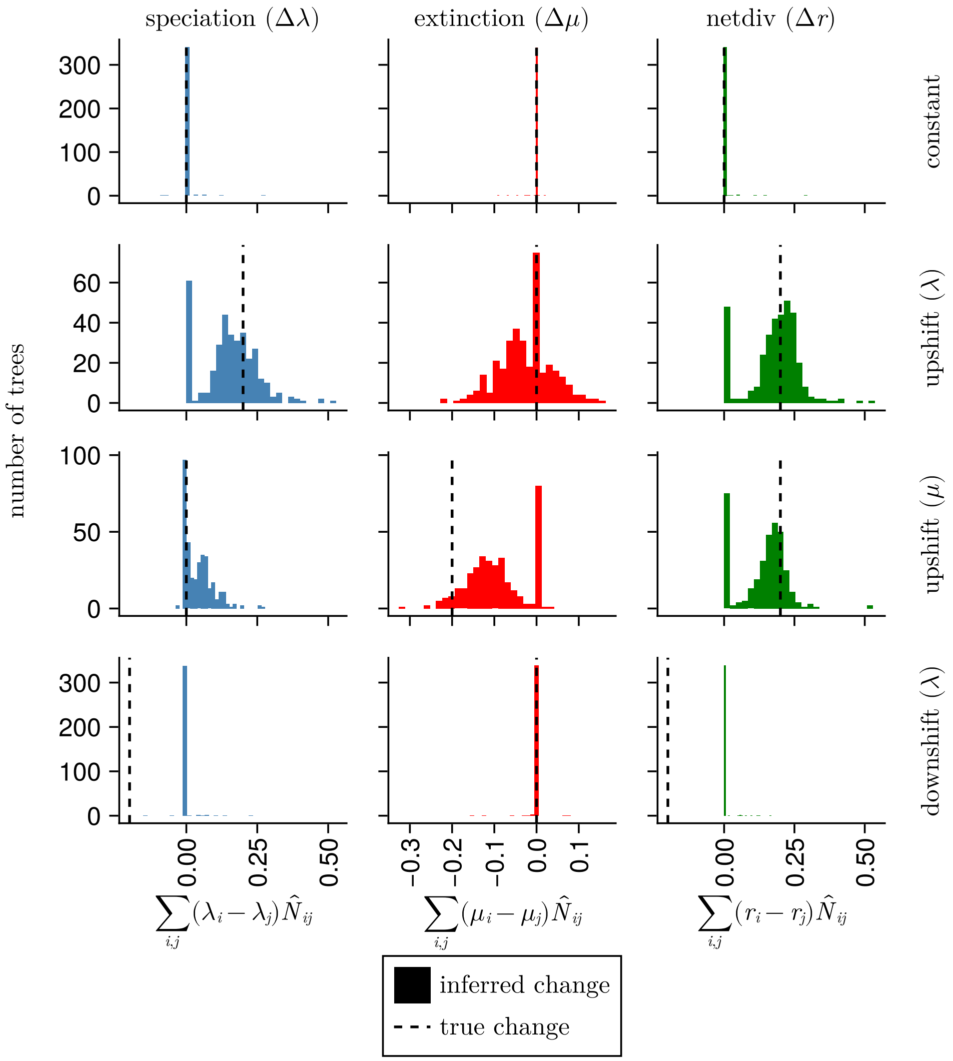

Figure S8: An assessment of the inferred change in branch-specific speciation, extinction and net-diversification rates, on simulated phylogenies with either no rate shift events (constant) or exactly one rate shift event (either upshift or downshift). We used the models in Table S2 to simulate various trees with various species richness. We simulated 350 backbone trees, which in combination with simulated in-group trees, we used to construct 350 upshift trees (due to an increase in λ), 350 upshift trees (due to a decrease in μ), and 350 downshift trees. Each data point in a histogram represents one tree. Each row represents the same set of trees.

S3 Validating the number of rate shift inferences using simulated phylogenies

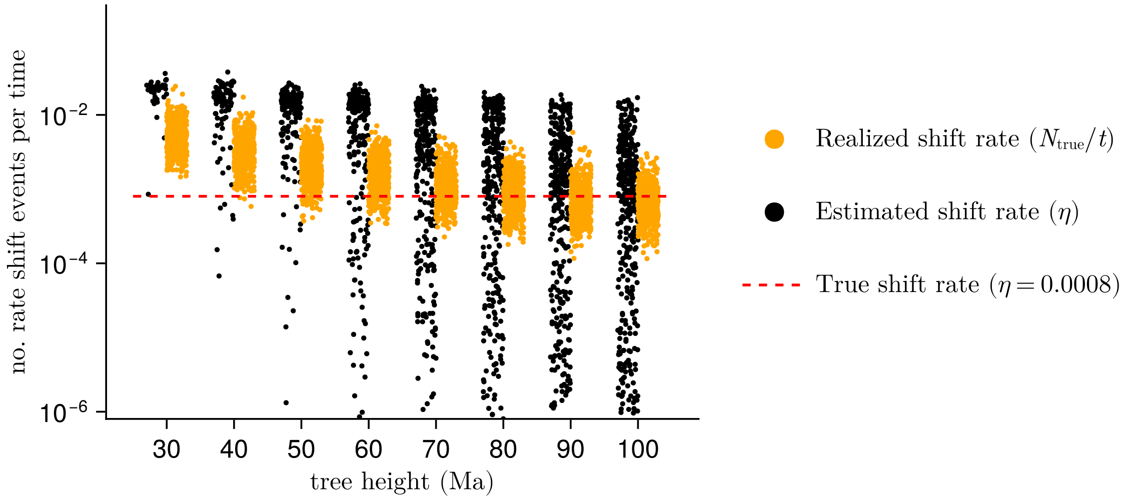

Figure S9: The shift rate (in units of rate shift events per time), estimated for several trees across a range of tree heights. Each dot represents one phylogeny. For each tree height in the range of 30 to 100 million years, we simulated 500 reconstructed trees and inferred the number of rate shifts. The position of the dots on the x-axis is jittered, i.e., moved randomly to the left or the right, for visibility purposes. The y-axis has been cut off at 10^{-6}, however there are many phylogenies with a smaller estimated shift rate (practically zero) that are not visible.

In the main text, we present analyses of empirical phylogenies, where the results indicate that there is a strong age-scaling effect for the number of rate shifts per time. We were concerned that this age-scaling effect was a result of a methodological artefact. To test whether this was an artefact, we set up a simulation study under a birth-death-shift model, where we can evaluate the performance of the birth-death-shift inference in a known scenario. Specifically, we simulated phylogenies under the following three-state model

$$\begin{aligned} \boldsymbol{\lambda} &= \quad [0.12, 0.21, 0.30] \\ \boldsymbol{\mu} &= \quad [0.08, 0.14, 0.20] \\ \eta &= \qquad\qquad 0.0008, \end{aligned} \tag{S5}$$

meaning that the relative extinction rate (μ/λ) was $2/3$, and the shift rate was selected such that we would get far fewer rate shift events than branching events. All simulations started in the category with the lowest net-diversification rate. We carefully hand-picked these rates in order to be able to simulate trees for a range of tree heights, without running into computational problems. Since extremely large trees are impractical, we set a maximum number of tips (50,000), and we rejected simulated trees that exceeded this threshold. In our experience, the hand-picked rates above generated trees with reasonable size for a range of tree heights (in the range of 30 to 100 million years, in increments of 10 myr), allowing us to test the inference of `Pesto` for a large number of tree replicates (500 per tree height), while keeping the simulation rejection rate at a reasonable level. This yielded 4000 simulated reconstructed trees in total, for which we also knew the true shift history along each branch.

Next, we inferred branch-specific diversification and branch-specific diversification rate events using `Pesto`. Specifically, specifically the following model setup

$$\begin{aligned} &(\hat{\lambda}, \hat{\mu}, \eta) && \text{estimated by ML under the birth-death-shift model} \\ &\vec{\lambda} && \text{six quantiles from LogNormal}(\log(\hat{\lambda}), \text{sd} = 0.587) \\ &\vec{\mu} && \text{six quantiles from LogNormal}(\log(\hat{\mu}), \text{sd} = 0.587) \\ &(\boldsymbol{\lambda}, \boldsymbol{\mu}) && \text{all pairwise combinations of } \vec{\lambda}, \vec{\mu}. \end{aligned} \tag{S6}$$

In other words, there are 36 rate categories (represented by $\boldsymbol{\lambda}, \boldsymbol{\mu}$). In Fig. S9, we see that the number of realized shifts per time (N_{true}/t) is higher than what is expected from the true shift rate (η). This is due to sampling biases, both since we i) conditioned on that the phylogenies must have at least one rate shift event, and ii) we conditioned on survival of the left and right subtrees descending from the root. When assessing the robustness of the method, we therefore investigated the estimation error as the difference between the estimates (η or N^*) and the realized number of rate shift events (N_{true}, based on the specific simulated rate shift histories).

In Fig. S10, we see that there is some estimation error for the shift rate (a) and the number of strongly inferred rate shifts events per time (b). Specifically, the shift rate is overestimated for young phylogenies, but is almost unbiased for older phylogenies. The number of strongly supported shift events per time (N^*/t) is underestimated for young phylogenies, however. This metric is also underestimated for older phylogenies, which we expect is due to that we are not able to infer downshifts reliably. In Fig. S11 we see the same data except plotted as a function of the number of tips. The patterns are broadly similar, in that the shift rate is overestimated, while the number of strongly supported rate shift events is underestimated, when considering phylogenies with few taxa.

The results tell us that the age scaling effect in the shift rate (η) could be partially explained by estimation error in young phylogenies. However, the age scaling effect in the number of strongly inferred rate shift events cannot be explained by estimation error in young phylogenies, as the direction of the bias is opposite to the empirical age scaling pattern. Thus, we do not believe that estimation error on its own can explain the age scaling pattern in the empirical analyses.

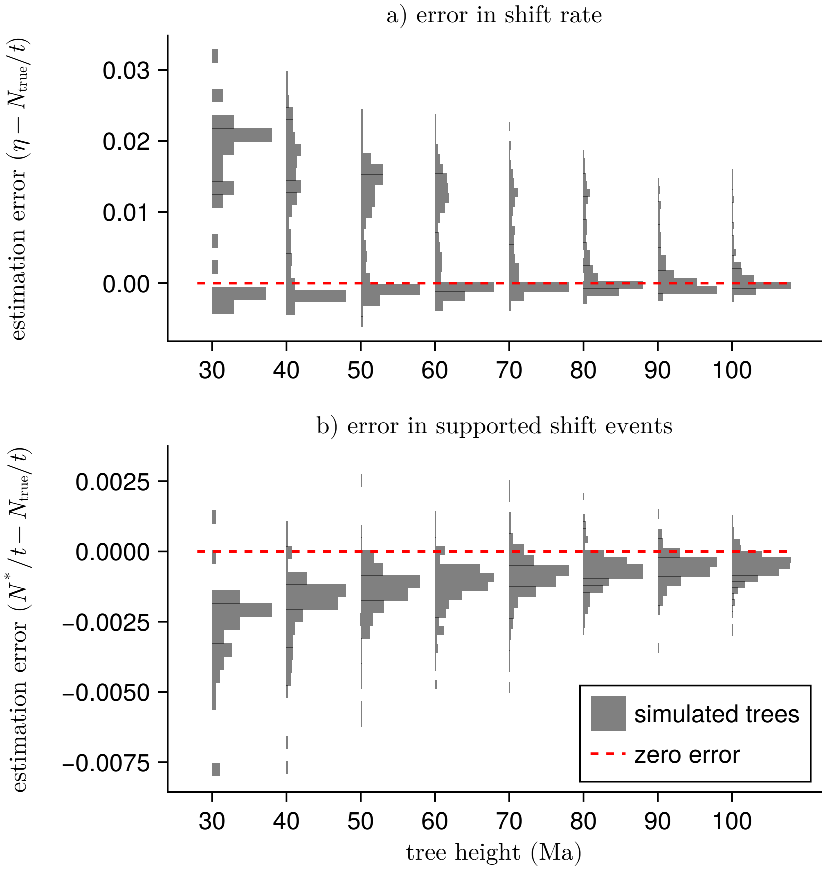

Figure S10: Estimation error in the number of rate shifts, as a function of tree height. The estimation error is the difference between the estimated value and the true value realized in the simulated trees. We used either the shift rate parameter (a) or the number of strongly supported branches per time (N^*/t) as the metric for the number of rate shift events. There are fewer trees for short tree heights (about 50 trees for 30 Ma), and more trees for longer tree heights (about 450 trees for 100 Ma). The shift rate η is overestimated for some young phylogenies, and it is approximately unbiased for old phylogenies.

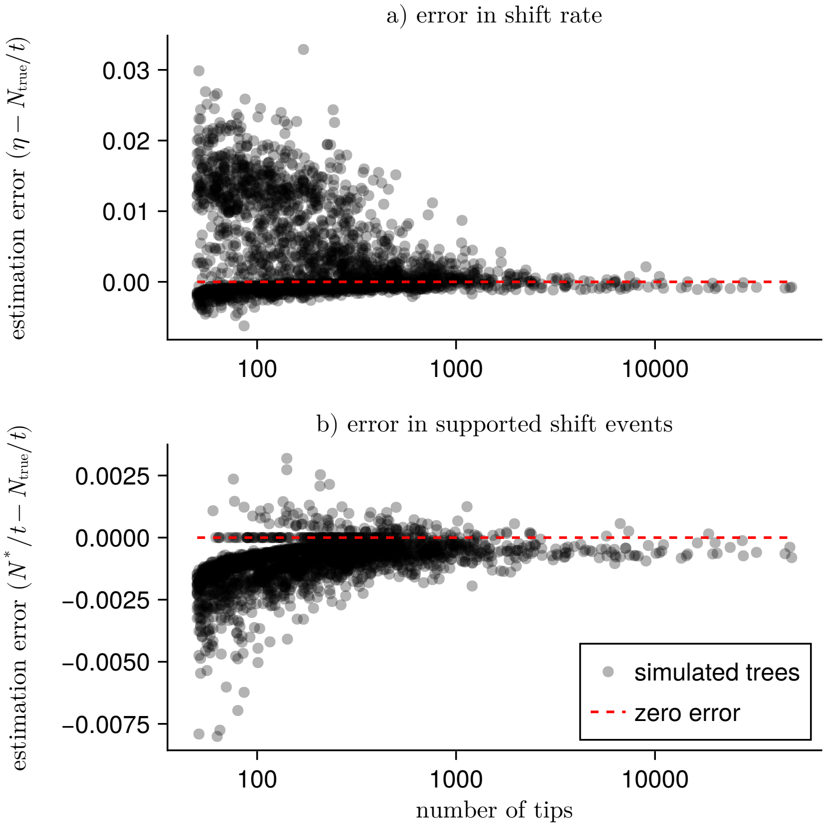

Figure S11: The same data as in Fig. S10 but as a function of the number of tips in the phylogeny. The shift rate η is overestimated for phylogenies with few tips, and the number of supported shifts per time (N^*/t) is underestimated for phylogenies with few tips.

S4 Within-phylogeny estimates of diversification shift rate

In the main text, we saw that there was an apparent age scaling effect of shift rate (η) with the clade age of the phylogeny. At first sight, it appears as if the diversification rate shifts more often for younger phylogenies than older phylogenies. We argued that this is a time averaging effect, which is caused by lack of information in the oldest parts of the phylogeny with which to infer shifts in the diversification rate. In the main text, we only compared the number of diversification rate shifts among phylogenies. In this section, we calculate and present posterior estimates of the number of diversification rate within phylogenies. If there is less evidence for diversification rate shifts in more ancient parts of the phylogenies, then we expect that the estimate for the number of rate shifts should be smaller in the past than at the present.

In order to calculate the number of rate shifts per time, we used our expression for the derivative of $\hat{N}$ with respect to time, i.e., $\frac{d\hat{N}_M}{dt(t)}(t)$ for a particular branch with index M. This quantity represents the branch-specific estimate for the instantaneous shift rate, i.e., the number of diversification rate shifts per time, specifically on branch M and at time t. Suppose that on a particular time t, say at the root of the phylogeny, t_{MRCA}, there are two active lineages in a set A. After the first branching event has occurred, there are three active lineages in the set B, and so on. In order to get a sense of the change across time, we discretized time from the root age of the phylogeny until the present. Next, we calculated the geometric mean of the shift rate across the number of active lineages like so

$$\text{mean shift rate} = \exp\left(\frac{1}{\sum_A 1} \sum_{M \in A} \log\left(\frac{d\hat{N}_M}{dt}(t)\right)\right), \tag{S7}$$

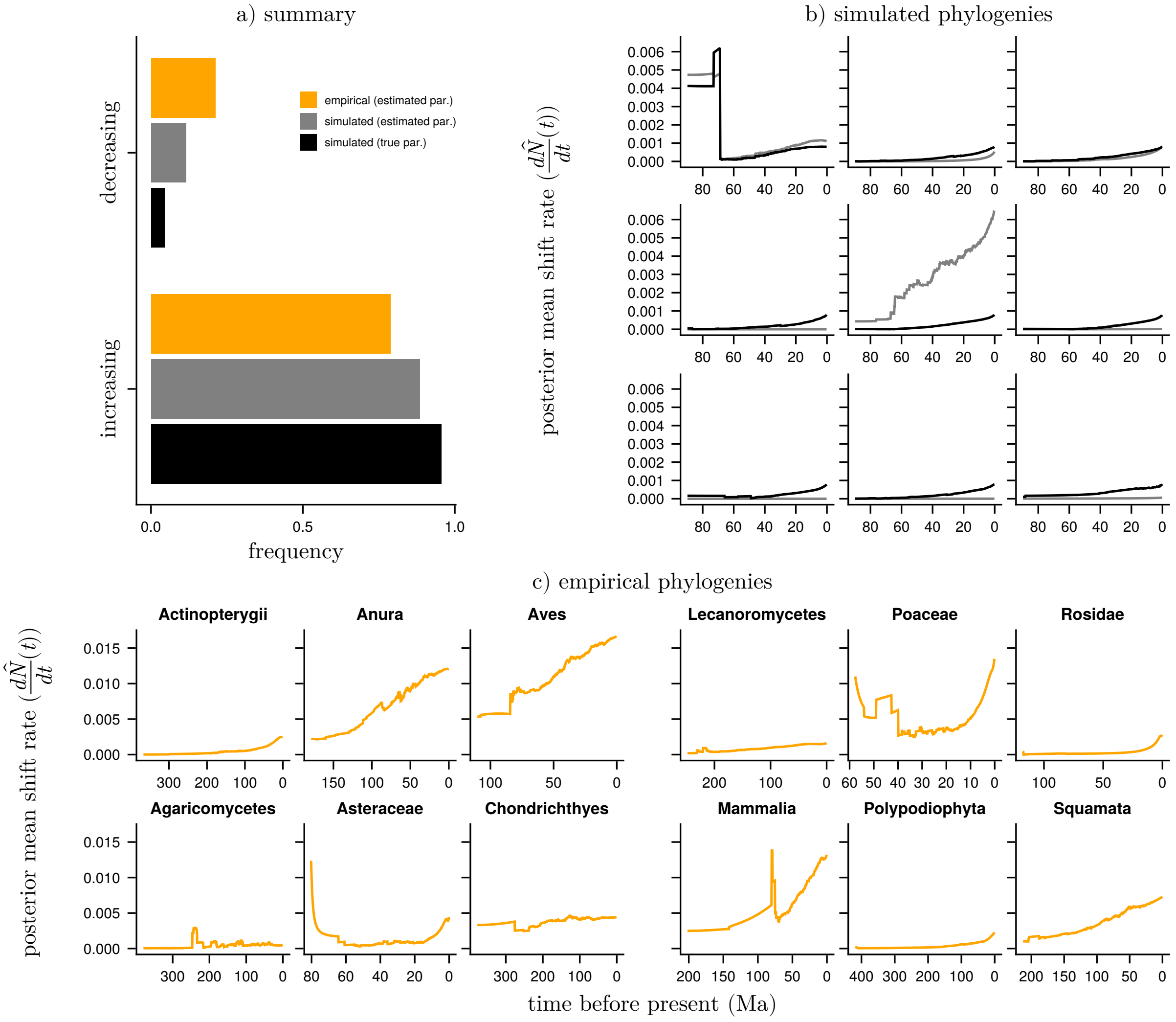

Figure S12: The posterior estimate for the shift rate, averaged (geometric mean) across the number of active lineages per time slice. a) represents a summary of whether the tempo of rate shifts overall increased or increased. If the most recent shift rate ($d\hat{N}/dt(t = 0)$) was greater than the shift rate at the time of the most-recent common ancestor (i.e., t_{MRCA}), then we counted the phylogeny as increasing. If not, we regarded the model as decreasing. b) represents 9 examples from simulated trees, with true parameters $\boldsymbol{r} = [0.04, 0.07, 0.10]$, $\epsilon = 2/3$ and $\eta = 0.0008$. The (in total 500) trees were simulated for a period of 90 Ma, see Section S3 for more details on the simulation setup. Panel c) shows 12 of the empirical phylogenies, specifically the ones featured Fig. 2. Note that the posterior estimate for the shift rate at the present (i.e., $dN/dt(t = 0)$) is equal to the estimate for the shift rate parameter η. In about 80–96% of the phylogenies, the shift rate was overall increasing through time.

for each time point t with its corresponding set of active lineages A. In other words, the mean posterior shift rate is an average of the instantaneous shift rate for all branches that were alive at a particular time in the past (and that survived until the present).

The results of this procedure is visualized in Fig. S12. In panel b) we show the mean shift rate through time for simulated phylogenies, whereas in c) we show the same for empirical phylogenies. Both panels b) and c) are a subsample of a greater number of phylogenies. When assessing each phylogeny individually, it appears at first as if the shift rate through time can change erratically, and it is different from phylogeny to phylogeny. Some shift histories may seem to match with prior knowledge in the group, for example in mammals the shifts were most prevalent briefly before the Cretaceous-Paleogene boundary, and in rosids there is a steady amount of rate shifts until very close to the present. Without specific knowledge of the particular group, however, it may be difficult to interpret what the rate shift history means.

One common pattern we noticed, was that the estimates of the rate shifts tended to increase through time. In order to summarize this, we computed whether the mean shift rate was higher at the present (i.t. $t = 0$) than at the root (i.e., $t = t_{\mathrm{MRCA}}$). This is shown in Fig. S12a, where we recorded between 80–96% phylogenies as having a shift rate estimate that was increasing through time within the phylogeny. This includes simulated phylogenies where the true shift rate is known, and is constant throughout all lineages and across time. For almost all (96%) of the simulated phylogenies we inferred a pattern of increasing shift rate through time.

In part this may seem reasonable, as if one were to consider a realized birth-death-shift history, then at the root of the tree there has not elapsed enough time for any rate shift event to occur, and therefore the estimate for the shift rate should be small. Interestingly, the mean shift rate at the present (i.e., $t = 0$) matches almost perfectly with the true shift rate when we set the parameter to the true value ($\eta = 0.0008$). However, the posterior estimate for the instantaneous shift rate tends to be smaller in older parts of the phylogeny. We argue that the branch-specific estimates of the shift-rate corroborates the pattern for the among-phylogeny estimates. As the effects of extinction hide the effects of rate shifts in older history more so than in recent history, this leads to an apparent but deceptive age scaling effect of the diversification shift rate.

S5 Assessing the age-scaling effect by analyzing subtrees of the ray-finned fish tree

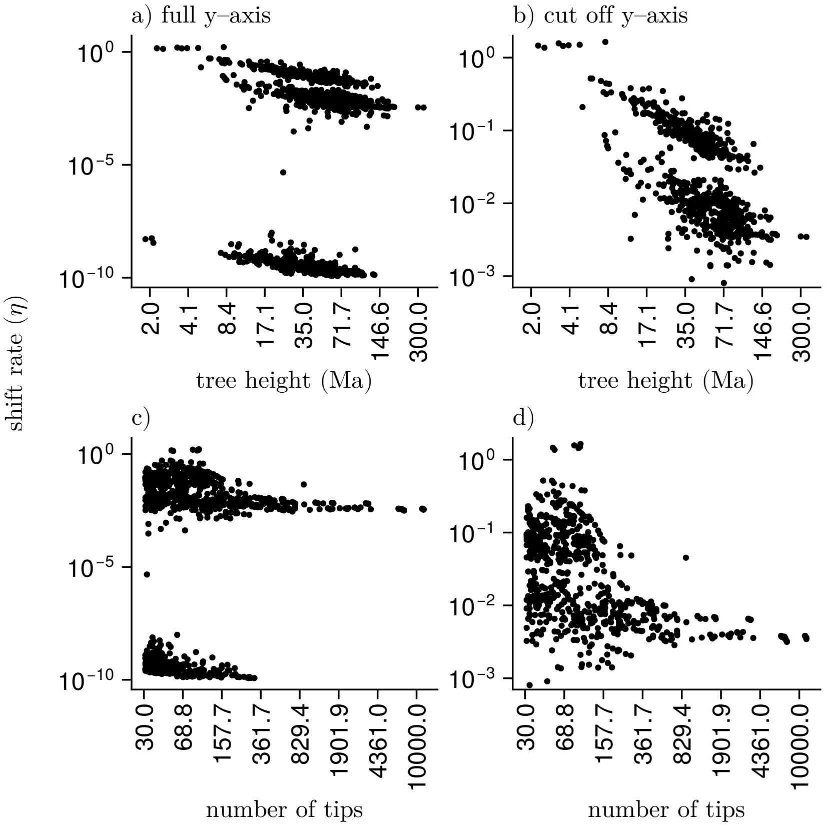

Figure S13: Each point represents a subtree in the ray-finned fish phylogeny (Rabosky et al., 2018). We included all subtrees that were represented by at least 30 species. Panels a) and b) depict the same scatter points, however in b) the y-axis has been truncated to remove the bottom portion of the points. We cut off the y-axis likewise in the bottom row, panels c) and d). Disregarding the clades for which we estimated none or negligibly small diversification rate variation (i.e., $\eta < 10^{-5}$), there is a negative scaling effect, in that young and species-rich clades exhibit far more diversification rate shifts per time than older and more established clades.

We also investigated the number of rate shift changes and its time-scaling effect on an empirical tree, the ray-finned fish phylogeny (Rabosky et al., 2018). To do so, we found all subtrees for the fish phylogeny that had at least 30 taxa represented at the present. This resulted in a total of 1035 subtrees. They ranged in tree height from approximately 2 million years (a cichlid subclade) to 330 million years, representing one of the earliest diverging lineages. For 310 subtrees, we estimated that there was none or negligibly small diversification rate variation (i.e., a shift rate of $\eta < 10^{-5}$). These trees were all represented by relatively few taxa ($<$ 360 no. taxa, Fig. S13).

The fish phylogeny is relatively large, and most splits starting from the root have many more than 30 living descendants. This means that many of the subtrees in Fig. S13 are also represented by their immediate left and right descendant clades. In other words, the dots in Fig. S13 are pseudoreplicates and are strongly correlated in a phylogenetic manner.

Young and species-rich clades like cichlids and icefishes appear to exhibit far more diversification rate shifts than older and more established clades. In particular, one clade showed an extreme amount of diversification rate shifts with $\eta \approx 20$. There are also a few other clades with a similar estimate for the number of rate shifts per time (Fig. S13, panel b). We argue that these extreme estimates are not robust, and arise due to how the phylogeny was reconstructed. Several internal branches in these clades have lengths of near zero,

as if a polytomy resolving technique had been used, and do not appear to be distributed across a range of branch lengths as would be expected under a birth-death (or birth-death-shift) process. Thus, while cichlids, icefishes and other highly-diversifying clades certainly exhibit significant diversification rate variation, we consider the specific estimates for branch-specific diversification rates, and diversification rate shifts in these clades to be unreliable.

Nevertheless, the big picture from Fig. S13 points to a strong negative relationship between the diversification shift rate (η) and the tree height, even within a single empirical phylogeny. Young and species-rich clades appear to have undergone far more diversification rate shifts per time than older and more established clades.

S6 Technical details on estimating the parameters

In the birth-death-shift model, there are three parameters that govern how the branching process behaves. These are $\hat{\lambda}, \hat{\mu}$, which control the mean of the speciation and extinction rate distributions, and the shift rate η which controls how often diversification rate shifts occur (Table S3).

$\hat{\lambda}$	the parameter controlling the mean of the speciation rate distribution
$\hat{\mu}$	the parameter controlling the mean of the extinction rate distribution
η	the parameter controlling how often diversification rate shifts occur
$\vec{\lambda}$	n quantiles from LogNormal(log($\hat{\lambda}$), sd=0.587)
$\vec{\mu}$	n quantiles from LogNormal(log($\hat{\mu}$), sd=0.587)
$(\boldsymbol{\lambda},\boldsymbol{\mu})$	all pairwise combinations of $\vec{\lambda}, \vec{\mu}$.

Table S3: Summary of parameters in the birth-death-shift model. The number of rate quantiles used is n, and the number of rate categories in $(\boldsymbol{\lambda},\boldsymbol{\mu})$ is $K = n^2$. The standard deviation of the log-normal distributions is set to 0.587 unless otherwise specified, which results in a log-normal distribution whose 2.5%–97.5% quantile spans one order of magnitude. The rate categories $(\boldsymbol{\lambda},\boldsymbol{\mu})$ are entirely determined by $\hat{\lambda}, \hat{\mu}$ and n.

In a simulation study, the parameters $(\hat{\lambda}, \hat{\mu}, \eta)$ are known without error. For empirical phylogenies, however, these are not known and must be estimated from the phylogeny. We chose to estimate the parameters by finding the parameter values that maximize the likelihood of the parameters given the phylogeny. We explored several ways of finding the maximum likelihood estimates using numerical optimization algorithms. In doing so, we discovered several scenarios in which the estimation procedure does not work well, or produced incoherent results. In a short list, these include:

(a) If the extinction rate ($\hat{\mu}$) is greater than the speciation rate ($\hat{\lambda}$)

(b) If the shift rate (η) is too high, for example greater than the speciation or extinction rate ($\hat{\lambda}$ or $\hat{\mu}$)

(c) If any of the parameters are too large

(d) If any of the parameters are too small

(e) If there are too few species in the phylogeny (e.g. < 25)

For this reason, we decided to impose several constraints on the optimization procedure. First, we imposed the constraint that $5\eta < \hat{\lambda} > \hat{\mu}$, by introducing three dummy variables, x_1, x_2 and x_3. We defined $\eta = x_1$, $\mu = x_2$, and $\lambda = \text{maximum}(5x_1, x_2) + x_3$, and optimizing over those transformed variables instead. This has an effect of imposing that the overall net-diversification is positive (remedying problem a), and that the shift rate is relatively small (remedying problem b). In the final rate categories $\boldsymbol{\lambda}, \boldsymbol{\mu}$, however, the net-diversification is allowed to be negative.

For the optimization algorithm, we decided to use Newton's method, since it is known to have fast (quadratic) convergence. Newton's method is a stepwise updating approach, where the next step $\vec{y}_{i+1}$ is updated as

$$\vec{y}_{i+1} = \vec{y}_i - kH(\vec{y}_i)^{-1}\nabla f(\vec{y}_i), \tag{S8}$$

where $\vec{y}_i$ is the current position, and k is some step size coefficient. $\nabla f(\vec{y}_i)$ is the gradient of the target function (i.e., the first derivatives of the negative log likelihood) evaluated at $\vec{y}_i$, and $H(\vec{y}_i)$ is the Hessian matrix (i.e., the second derivatives of the negative log likelihood) evaluated at $\vec{y}_i$. $\vec{y}_0$ is some starting point. We used automatic differentiation to compute the derivatives (Revels et al., 2016), and we used the implementation of Mogensen and Riseth (2018) for Newton's method. Newton's method is more computationally expensive than gradient-free methods or gradient descent per iteration, as it requires one to compute and invert the Hessian matrix. The extra computational cost is in practice not a problem, as it is outweighed by the fast convergence of Newton's method, and we typically need few (< 50) iterations before convergence is reached. One challenge with Newton's method is, however, that it only works for a problem where the parameters

are unconstrained, i.e., $\vec{y}$ must be allowed to vary between $-\infty$ and $+\infty$. As a fix, we re-transformed our parameters x_1, x_2, x_3 using a logistic function with soft boundaries:

$$g(y) = \frac{\mathrm{U} - \mathrm{L}}{1 + e^{-s(y-\mathrm{M}))}} + \mathrm{L}. \tag{S9}$$

Here, U is the upper limit, L is the lower limit, $M = (\mathrm{U} + \mathrm{L})/2$ is the midpoint, and $s = 1/2$ is a steepness coefficient. The inverse function is

$$h(x) = \mathrm{M} - \frac{1}{s} \log\left(\frac{\mathrm{U} - \mathrm{L}}{x - \mathrm{L}} - 1\right), \tag{S10}$$

and we used lower limits $\mathrm{L} \in \{10^{-8}, 10^{-4}, 10^{-4}\}$, upper limits $\mathrm{U} \in \{0.3, 1.0, 1.0\}$ for x_1, x_2, x_3, respectively. Since this transform effectively maps our constrained parameters to an unconstrained space, we can use Newton's method to optimize for the maximum likelihood parameters. By imposing lower and upper limits for our parameters, we remedy problems c) and d).

The choice of starting point $\vec{y}_0$ also has an impact on the optimization procedure. We observed that, if different starting points $\vec{y}_0$ are chosen, the optimization routine will find different (local) maximum likelihood regions. In other words, if the procedure is run a few times, it may reach two or more different solutions. Although the log-likelihood values may not be too different, the inferred position, size and number of rate shift events on the phylogeny may vary. For example, one may get a solution A which represents one large rate shift on one branch, and a solution B which represents several smaller diversification rate shifts on several branches in different parts of the phylogeny. When running the analyses on an empirical phylogeny, we therefore strongly recommend to repeat the inference procedure several times, and to pick the solution that maximizes the (global) likelihood.

In the inference procedure, we opted for using a random starting position $\vec{x}$. First, we estimated the net-diversification rate ($r_{\mathrm{CBD}} > 0$) and the extinction rate ($\mu_{\mathrm{CBD}} > 0$) under the constant-rate (i.e., lineage-homogeneous) birth-death model (CBD). With these estimates, we set up three distributions:

$$\begin{aligned} d_1 &\sim \mathrm{LogNormal}(\log(0.01), \mathrm{sd} = 0.5) \\ d_2 &\sim \mathrm{LogNormal}(\log(\mu_{\mathrm{CBD}}), \mathrm{sd} = 0.5) \\ d_3 &\sim \mathrm{LogNormal}(\log(r_{\mathrm{CBD}}), \mathrm{sd} = 0.5). \end{aligned} \tag{S11}$$

We drew random starting points x_1, x_2, x_3 from the distributions d_1, d_2, d_3, which we transformed to the parameters y_1, y_2, y_3 using Eq. (S10).

For problem e), we simply decided not to assess the birth-death-shift model for phylogenies with fewer than 50 taxa. This is not a big problem, as inferences made from such phylogenies will anyways be unreliable, and it is perhaps better to make the simplifying assumption that there is no among-lineage rate variation.

S7 Behaviour with higher rate class discretization

In the main text, we noted that, as the number of rate classes increases, the estimate for i) speciation rate shifts and ii) extinction rate shifts converges to zero, and iii) the estimate for joint speciation and extinction rate shift converges to the total. When a rate shift event happens in the birth-death-shift model [with discretized base distributions], there are three possible outcomes:

1. the speciation rate changes (with probability $(n-1)/(n^2-1)$),
2. the extinction rate changes (with probability $(n-1)/(n^2-1)$),
3. the speciation and extinction rate change simultaneously (with probability $((n^2-2n+1)/(n^2-1))$.

These events are depicted in Fig. S14, panels a, b, c, and colored orange, teal and gray, respectively. The area of panels a, b, d corresponds to the probability of the event type. When the number of rate classes increases from 5 to 7 (panels a to c), it becomes evident that there are more opportunities for a joint change to happen (gray color), as opposed to single changes to happen (orange or teal). As the number of rate classes approaches a large number, the number of single rate change events (speciation or extinction) approaches zero, and the joint change events begin to dominate (panel d).

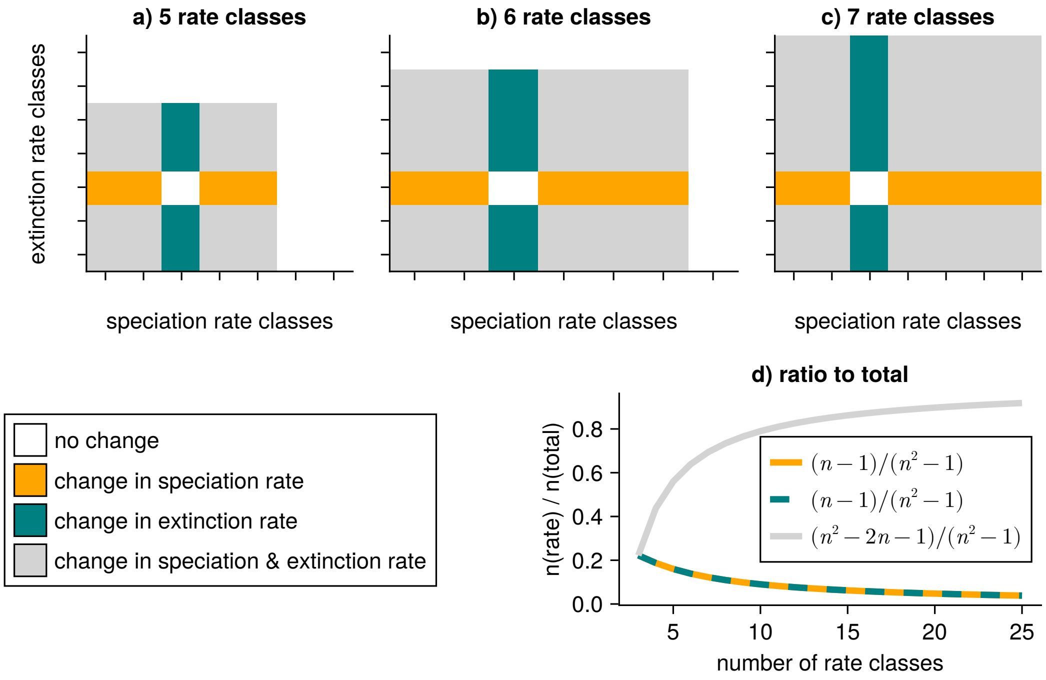

Figure S14: Visualization of how the type of diversification rate shifts (in speciation, extinction or joint shifts) are assumed to happen, when the number of rate class discretizations increase. The white square is the "ancestral rate category", and the colored squares represent the different outcomes of the diversification rate shift event. The figure could be re-drawn with the "ancestral rate category" (white square) placed in any other position, without changing the meaning of the figure. This distribution of different types of rate shift events is conditional on that a rate shift event (of unknown type) has already occurred; i.e., the parameter controlling the number of rate shift events per time (η) is irrelevant for the figure.

References

Allio, R., Nabholz, B., Wanke, S., Chomicki, G., Pérez-Escobar, O. A., Cotton, A. M., Clamens, A.-L., Kergoat, G. J., Sperling, F. A., and Condamine, F. L. (2021). Genome-wide macroevolutionary signatures of key innovations in butterflies colonizing new host plants. *Nature Communications*, 12(1):354.

Álvarez-Carretero, S., Tamuri, A. U., Battini, M., Nascimento, F. F., Carlisle, E., Asher, R. J., Yang, Z., Donoghue, P. C., and Dos Reis, M. (2022). A species-level timeline of mammal evolution integrating phylogenomic data. *Nature*, 602(7896):263–267.

Bank, S. and Bradler, S. (2022). A second view on the evolution of flight in stick and leaf insects (phasmatodea). *BMC ecology and evolution*, 22(1):62.

Henríquez-Piskulich, P., Hugall, A. F., and Stuart-Fox, D. (2024). A supermatrix phylogeny of the world's bees (hymenoptera: Anthophila). *Molecular Phylogenetics and Evolution*, 190:107963.

Höhna, S. (2015). The time-dependent reconstructed evolutionary process with a key-role for mass-extinction events. *Journal of Theoretical Biology*, 380:321–331.

Höhna, S., May, M. R., and Moore, B. R. (2016). TESS: an R package for efficiently simulating phylogenetic trees and performing Bayesian inference of lineage diversification rates. *Bioinformatics*, 32(5):789–791.

Jetz, W., Thomas, G. H., Joy, J. B., Hartmann, K., and Mooers, A. Ø. (2012). The global diversity of birds in space and time. *Nature*, 491(7424):444–448.

Kawahara, A. Y., Storer, C., Carvalho, A. P. S., Plotkin, D. M., Condamine, F. L., Braga, M. P., Ellis, E. A., St Laurent, R. A., Li, X., Barve, V., et al. (2023). A global phylogeny of butterflies reveals their evolutionary history, ancestral hosts and biogeographic origins. *Nature ecology & evolution*, 7(6):903–913.

Kopperud, B. T. and Höhna, S. (2025). Phylogenetic Estimation of Branch-Specific Shifts in the Tempo of Origination. *Systematic Biology*.

Kriebel, R., Drew, B. T., Drummond, C. P., González-Gallegos, J. G., Celep, F., Mahdjoub, M. M., Rose, J. P., Xiang, C.-L., Hu, G.-X., Walker, J. B., et al. (2019). Tracking temporal shifts in area, biomes, and pollinators in the radiation of salvia (sages) across continents: leveraging anchored hybrid enrichment and targeted sequence data. *American Journal of Botany*, 106(4):573–597.

Leslie, A. B., Beaulieu, J., Holman, G., Campbell, C. S., Mei, W., Raubeson, L. R., and Mathews, S. (2018). An overview of extant conifer evolution from the perspective of the fossil record. *American journal of botany*, 105(9):1531–1544.

Letsch, H., Gottsberger, B., and Ware, J. L. (2016). Not going with the flow: a comprehensive time-calibrated phylogeny of dragonflies (anisoptera: Odonata: Insecta) provides evidence for the role of lentic habitats on diversification. *Molecular ecology*, 25(6):1340–1353.

Liu, J., Lindstrom, A. J., Nagalingum, N. S., Wiens, J. J., and Gong, X. (2021). Testing the causes of richness patterns in the paleotropics: time and diversification in cycads (cycadaceae). *Ecography*, 44(11):1606–1618.

Mogensen, P. K. and Riseth, A. N. (2018). Optim: A mathematical optimization package for Julia. *Journal of Open Source Software*, 3(24):615.

Mortimer, S. M., Boyko, J., Beaulieu, J. M., and Tank, D. C. (2022). Synthesizing existing phylogenetic data to advance phylogenetic research in orobanchaceae. *Systematic Botany*, 47(2):533–544.

Mulder, C. (2003). Aristolochiaceae. *Review of Palaeobotany and Palynology*, 123(1-2):47–55.

Nelsen, M. P., Lücking, R., Boyce, C. K., Lumbsch, H. T., and Ree, R. H. (2020). The macroevolutionary dynamics of symbiotic and phenotypic diversification in lichens. *Proceedings of the National Academy of Sciences*, 117(35):21495–21503.

Nitta, J. H., Schuettpelz, E., Ramírez-Barahona, S., and Iwasaki, W. (2022). An open and continuously updated fern tree of life. *Frontiers in Plant Science*, 13:909768.

Palazzesi, L., Hidalgo, O., Barreda, V. D., Forest, F., and Höhna, S. (2022). The rise of grasslands is linked to atmospheric CO_2 decline in the late Paleogene. *Nature Communications*, 13(1):293.

Portik, D. M., Streicher, J. W., and Wiens, J. J. (2023). Frog phylogeny: a time-calibrated, species-level tree based on hundreds of loci and 5,242 species. *Molecular Phylogenetics and Evolution*, 188:107907.

Quintero, I., Suchard, M. A., and Jetz, W. (2022). Macroevolutionary dynamics of climatic niche space. *Proceedings of the Royal Society B*, 289(1975):20220091.

Rabosky, D. L., Chang, J., Cowman, P. F., Sallan, L., Friedman, M., Kaschner, K., Garilao, C., Near, T. J., Coll, M., Alfaro, M. E., et al. (2018). An inverse latitudinal gradient in speciation rate for marine fishes. *Nature*, 559(7714):392–395.

Revels, J., Lubin, M., and Papamarkou, T. (2016). Forward-mode automatic differentiation in Julia. *arXiv*.

Rose, J. P., Kleist, T. J., Löfstrand, S. D., Drew, B. T., Schönenberger, J., and Sytsma, K. J. (2018). Phylogeny, historical biogeography, and diversification of angiosperm order ericales suggest ancient neotropical and east asian connections. *Molecular Phylogenetics and Evolution*, 122:59–79.

Serrano-Serrano, M. L., Rolland, J., Clark, J. L., Salamin, N., and Perret, M. (2017). Hummingbird pollination and the diversification of angiosperms: an old and successful association in gesneriaceae. *Proceedings of the Royal Society B: Biological Sciences*, 284(1852):20162816.

Smith, S. A., Brown, J. W., Yang, Y., Bruenn, R., Drummond, C. P., Brockington, S. F., Walker, J. F., Last, N., Douglas, N. A., and Moore, M. J. (2018). Disparity, diversity, and duplications in the caryophyllales. *New Phytologist*, 217(2):836–854.

Spriggs, E. L., Christin, P.-A., and Edwards, E. J. (2014). C4 photosynthesis promoted species diversification during the miocene grassland expansion. *PloS one*, 9(5):e97722.

Stein, R. W., Mull, C. G., Kuhn, T. S., Aschliman, N. C., Davidson, L. N., Joy, J. B., Smith, G. J., Dulvy, N. K., and Mooers, A. O. (2018). Global priorities for conserving the evolutionary history of sharks, rays and chimaeras. *Nature ecology & evolution*, 2(2):288–298.

Sun, M., Folk, R. A., Gitzendanner, M. A., Soltis, P. S., Chen, Z., Soltis, D. E., and Guralnick, R. P. (2020). Recent accelerated diversification in rosids occurred outside the tropics. *Nature communications*, 11(1):3333.

Thomas, S. K., Liu, X., Du, Z.-Y., Dong, Y., Cummings, A., Pokorny, L., Xiang, Q.-Y., and Leebens-Mack, J. H. (2021). Comprehending cornales: phylogenetic reconstruction of the order using the angiosperms353 probe set. *American Journal of Botany*, 108(7):1112–1121.

Title, P. O., Singhal, S., Grundler, M. C., Costa, G. C., Pyron, R. A., Colston, T. J., Grundler, M. R., Prates, I., Stepanova, N., Jones, M. E., et al. (2024). The macroevolutionary singularity of snakes. *Science*, 383(6685):918–923.

Upham, N., C, B., J, W., Becker, M., Handika, H., Zijlstra, J., and Huckaby, D. (2024). Mammal diversity database (version 1.13). `https://doi.org/10.5281/zenodo.10595931`. Accessed: 2024-11-27.

Varga, T., Krizsán, K., Földi, C., Dima, B., Sánchez-García, M., Sánchez-Ramírez, S., Szöllősi, G. J., Szarkándi, J. G., Papp, V., Albert, L., et al. (2019). Megaphylogeny resolves global patterns of mushroom evolution. *Nature Ecology & Evolution*, 3(4):668–678.

Willink, B., Ware, J. L., and Svensson, E. I. (2024). Tropical origin, global diversification, and dispersal in the pond damselflies (coenagrionoidea) revealed by a new molecular phylogeny. *Systematic Biology*, 73(2):290–307.
